# The Identification and Genetic Characterization of Parechovirus Infection Among Pediatric Patients With Wide Clinical Spectrum in Chongqing, China

**DOI:** 10.3389/fmicb.2021.709849

**Published:** 2021-09-14

**Authors:** Xiao-Ai Zhang, Rui-Qiu Zhao, Jin-Jin Chen, Yang Yuan, Xiang Tang, Zi-Wei Zhou, Luo Ren, Qin-Bin Lu, Yu-Na Wang, Hai-Yang Zhang, Pan-He Zhang, Li-Qun Fang, Hai-Sheng Zhou, En-Mei Liu, Hong-Mei Xu, Wei Liu

**Affiliations:** ^1^State Key Laboratory of Pathogen and Biosecurity, Beijing Institute of Microbiology and Epidemiology, Beijing, China; ^2^Children’s Hospital of Chongqing Medical University, Chongqing, China; ^3^Department of Laboratorial Science and Technology, School of Public Health, Peking University, Beijing, China; ^4^Key Laboratory of Dermatology, Anhui Medical University, Hefei, China; ^5^Department of Biochemistry and Molecular Biology, Anhui Medical University, Hefei, China; ^6^Beijing Key Laboratory of Vector Borne and Natural Focus Infectious Diseases, Beijing, China

**Keywords:** parechovirus, epidemiology, respiratory infection, acute diarrhea, hand foot and mouth disease

## Abstract

Human parechoviruses (HPeVs) are important causes of infection in children. However, without a comprehensive and persistent surveillance, the epidemiology and clinical features of HPeV infection remain ambiguous. We performed a hospital-based surveillance study among three groups of pediatric patients with acute respiratory infection (Group 1), acute diarrhea (Group 2), and hand, foot and mouth disease (Group 3) in Chongqing, China, from 2009 to 2015. Among 10,212 tested patients, 707 (6.92%) were positive for HPeV, with the positive rates differing significantly among three groups (Group 1, 3.43%; Group 2, 14.94%; Group 3, 3.55%; *P* < 0.001). The co-infection with other pathogens was detected in 75.2% (531/707) of HPeV-positive patients. Significant negative interaction between HPeV and Parainfluenza virus (PIV) (*P* = 0.046, OR = 0.59, 95% CI = 0.34–0.98) and positive interactions between HPeV and Enterovirus (EV) (*P* = 0.015, OR = 2.28, 95% CI = 1.23–4.73) were identified. Among 707 HPeV-positive patients, 592 (83.73%) were successfully sequenced, and 10 genotypes were identified, with HPeV1 (*n* = 396), HPeV4 (*n* = 86), and HPeV3 (*n* = 46) as the most frequently seen. The proportion of genotypes differed among three groups (*P* < 0.001), with HPeV1 and HPeV4 overrepresented in Group 2 and HPeV6 overrepresented in Group 3. The spatial patterns of HPeV genotypes disclosed more close clustering of the currently sequenced strains than those from other countries/regions, although they were indeed mixed. Three main genotypes (HPeV1, HPeV3, and HPeV4) had shown distinct seasonal peaks, highlighting a bi-annual cycle of all HpeV and two genotypes (HPeV 1 and HPeV 4) with peaks in odd-numbered years and with peaks in even-numbered years HPeV3. Significantly higher HPeV1 viral loads were associated with severe diarrhea in Group 2 (*P* = 0.044), while associated with HPeV single infection than HPeV-EV coinfection among HFMD patients (*P* = 0.001). It’s concluded that HPeV infection was correlated with wide clinical spectrum in pediatric patients with a high variety of genotypes determined. Still no clinical significance can be confirmed, which warranted more molecular surveillance in the future.

## Introduction

Human parechoviruses (HPeVs) are small, non-enveloped RNA viruses closely related to enteroviruses (EVs) in the *Picornaviridae* family. The *Parechovirus* genus currently contains 6 species, Parechovirus A to F.^1^ Among them Parechovirus A is the only species of *Parechovirus* genus which infects humans (de Crom et al., 2016b). Similar to Enterovirus, Parechovirus A causes frequent infection in children, presenting with a wide clinical spectrum that spanned from asymptomatic infection, mild illness, to severe diseases (Cordey et al., 2015). To date, a total of 19 HPeV genotypes (HPeV1–HPeV19) had been identified, with their causative illness also differing among the determined types (Shakeel et al., 2017). HPeV genotypes 1, 3, and 6 are most commonly associated with human disease (de Crom et al., 2016b; Olijve et al., 2018). Of particular interest is HPeV3, which has been suggested as a leading cause of sepsis-like illness and central nervous system infection in neonates and young infants (Boivin et al., 2005; Olijve et al., 2018). The circulation of HPeV in China has been previously studied with small sample size and short duration, and mostly limited to diarrhea (Lin et al., 2008; Shan et al., 2009; Li et al., 2010; Cai et al., 2014). Without a comprehensive and persistent surveillance, the epidemiological and genetic characteristics of HPeV infection remain ambiguous. The present study was designed to systematically investigate the prevalence, epidemiological feature, and genetic diversity of HPeV in pediatric patients in China through a long-lasting surveillance period in three groups of patients.

## Materials and Methods

### Patient Recruitment

During 2009–2015, a hospital-based surveillance study was performed at the Children’s hospital, Chongqing Medical University (CHCMU), the largest children’s hospital in southwestern China, serving patients with a wide geographic range across central and western China. Since 2009, a laboratory surveillance has been performed to detect the viral infections in three groups of patients, i.e., with acute respiratory tract infection (ARTI), acute diarrhea, or hand, foot and mouth disease (HFMD). The ARTI was defined on the basis of acute disease onset with symptoms of cough, rhinorrhea and/or dyspnea, and/or fever of > 37.5°C. Fever caused by known chronic medical conditions was excluded. The pneumonia was defined by the presence of patchy alveolar opacities on chest radiographs, in addition to symptoms of cough, dyspnea (lower chest wall indrawing), or tachypnea. Hospitalized patients with moderate, severe, and critical severe pneumonia were diagnosed as severe ARTI. Diarrhea was defined as > 3 loose stools in the previous 24 h. Patients who had confirmed inflammatory bowel disease, celiac disease, cystic fibrosis, food intolerance, or patients who had any apparent clinical respiratory signs or symptoms were excluded. Severe diarrhea was defined as ≥ 5 loose stools in the previous 24 h. HFMD were diagnosed according to the guidelines released by the Ministry of Health of the People’s Republic of China.^2^ Severe HFMD was defined if one of the following complications were additionally presented: encephalitis, meningitis, AFP, or cardiorespiratory failure. Before the surveillance was started, the staff that participated in the study had undergone training to be qualified for recruiting patients, sample collection, and test following the standard operating procedures (SOP) developed by Beijing Institute of Microbiology and Epidemiology. The tested specimen included nasopharyngeal swab (NPAs) for patients with ARTI and stool for patients with diarrhea or HFMD. All the samples were required to be collected on patients’ admission into the hospital. A standardized questionnaire was used to obtain the patients’ information from medical records, including demographic data, underlying medical conditions, symptoms and signs, routine blood test results, radiographic findings, and disease outcome. This study was performed with the approval of the Ethics Review Committee of CHCMU, and in accordance with the approved SOP throughout the years. At recruitment, written informed consent was obtained from all guardians of participants.

### Laboratory Detection

Viral RNA/DNA was isolated from stool or swab samples using QIAamp Viral RNA Mini Kit (Qiagen, Germantown, MD, United States) according to the manufacturer’s instructions. All samples were screened for HPeV using real-time RT-PCR assay targeting the 5′untranslated region (UTR) as previously described (Benschop et al., 2008). All HPeV positive samples were amplified for the viral protein (VP) 1 or VP3/VP1 genes using nested RT-PCR or touch-down RT-PCR approach for genotyping as previously described (Benschop et al., 2006; Harvala et al., 2008; Cristanziano et al., 2015; Brouwer et al., 2019; Pietsch and Liebert, 2019; Supplementary Table 1). All the amplicons were subject to sequencing and BLAST analysis.

For all the NPA samples collected from patients with ARTI, influenza virus (IFV), metapneumovirus (MPV), respiratory syncytial virus (RSV), parainfluenza virus (PIV), human rhinovirus (HRV), and coronavirus (CoV) were tested by reverse transcriptase polymerase chain reaction (RT-PCR), and human bocavirus (HBoV) and human adenovirus (HadV) were tested by performing polymerase chain reaction (PCR) as described previously (Xu et al., 2000; Gunson et al., 2005; Tiveljung-Lindell et al., 2009). Five viral enteric pathogens were tested for the stool specimens collected from patients with acute diarrhea. Rotavirus A antigen was tested by enzyme-linked immunosorbent assay. Testing of norovirus (NoV), HadV, astrovirus (AstV), and sapovirus (SaV) was performed by PCR or RT-PCR (Rohayem et al., 2004; Qu et al., 2007; Stals et al., 2009). The stool samples from HFMD patients had been simultaneously tested for enterovirus (EV) by using real-time RT-PCR (Verstrepen et al., 2002). All steps of the nucleic acid extraction and RT-PCR/PCR test were conducted in parallel with positive and negative controls.

The HpeV viral load was determined by using the same primers and probes as for detection. Plasmids containing known copy number of amplification targets were applied in real-time RT-PCR test to generate a standard curve for quantification of test samples. Positive and negative controls were included. The copy number of virus expressed as copies/mL was determined by comparison with a serially diluted plasmid standard of known concentration. All samples were quantified in at least duplicate wells.

### Phylogenetic Analysis

The sequences obtained from the study were submitted to NCBI with the GenBank Accession Numbers MT700562-MT700938 and MT702393-MT702579. Genomic sequences were assembled using Lasergene’s DNA SeqMan software (version 7.1.0, DNA Star Inc., Madison, WI, United States). The phylogenetic trees were constructed with maximum likelihood method with 1,000 replicates using MEGA 7.0.

### Statistical Analysis

Descriptive statistics were performed for categorical variables as frequencies and proportions or rates, and continuous variables were expressed as medians and interquartile ranges. The non-parametric test with Mann-Whitney *U*-test method was used to compare the differences of continuous variables between different groups. Pearson chi-square test or Fisher exact test was performed to compare the differences of categorical variables. Logistic regression models were used to determine the association between clinical characteristics and HPeV infection. Odds ratio (OR) and its 95% CI were estimated using maximum likelihood method. The viral loads were estimated for their association with age, sex, disease severity, and single/co-infection by multiple linear regression analysis. Statistical analysis was performed using R software (version 3.5.3). The interactions between HPeV and each of the other detected viruses was explored at the scale of individual hosts as previously described (Nickbakhsh et al., 2019).

## Results

### Detection of HPeV in Three Groups of Patients

**FIGURE 1:**
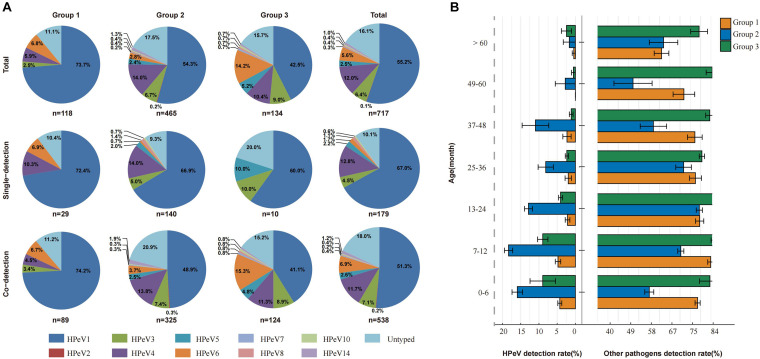
The single infection and co-infection pattern, phylogenetic analysis of all the HPeV, and the age specific pattern of HPeV among three groups of pediatric patients. **(A)** HPeV single infection and co-infection rate and co-infection pattern between each HPeV genotype and the predominant viruses among three groups of pediatric patients. There were 10 patients coinfected with two genotypes of HPeVs, including HPeV1-HPeV4 coinfection in 7 patients in Group 2, HPeV1-HPeV4 coinfection in 1 patient in Group 3, HPeV3-HPeV6 coinfection in 1 patient in Group 2, and HPeV1-HPeV5 coinfection in 1 patient in Group 3. **(B)** The age specific pattern of HPeV and other viruses infection among three groups of pediatric patients. Group 1: patients with acute respiratory tract infection (ARTI), Group 2: patients with acute diarrhea, Group 3: patients with hand foot and mouth disease (HFMD).

Altogether 10,212 pediatric patients, including 3,438 hospitalized patients with ARTI (Group 1), 3,059 with acute diarrhea (Group 2), and 3,715 with HFMD (Group 3), met the inclusion criteria and were recruited into the study (Supplementary Table 2). The age of Group 3 was significantly higher than that of Group 1 and Group 2 (*P* < 0.001), while sex was comparable among three groups. In total, 6.92% (707/10212) of patients were positive for HPeV (Table 1 and Figure 1A), with the positive rates differing significantly among three groups (3.43%, 118/3,438 for Group 1, 14.94%, 457/3,059 for Group 2, and 3.55%, 132/3,715 for Group 3, *P* < 0.001). The co-infections were counted by positive test for HPeV and any one of the other tested viruses. Both rates of single infection (1.72%, 176/10,212) and co-infection involving HPeV (5.20%, 531/10,212) differed among three groups, with the highest co-infection rate/single infection rate ratio observed for Group 3 (3.28% vs. 0.27%, *P* < 0.001; Table 1).

**TABLE 1:** The detection and genotyping of HPeVs in three cohorts of pediatric patients.

Characteristics	Total	Group 1^$^	Group 2^$^	Group 3^$^	*P*-value
	(*n* = 10212)	(*n* = 3438)	(*n* = 3059)	(*n* = 3715)	
**All HpeV infected patients**	707(6.92)	118 (3.43)	457 (14.94)	132 (3.55)	<0.001^$^
Single infection	176 (1.72)	29 (0.84)	137 (4.48)	10 (0.27)	<0.001^$^
Coinfection	531 (5.20)	89 (2.59)	320 (10.46)	122 (3.28)	
**Age groups (months)**					<0.001^#^
0–6	166 (7.97)	59 (4.27)	101 (15.93)	6 (8.96)	
7–12	224 (9.47)	38 (4.77)	172 (14.74)	34 (8.46)	
13–24	152 (5.56)	11 (2.15)	92 (10.03)	49 (3.76)	
25–36	42 (2.59)	5 (1.96)	13 (7.56)	24 (2.01)	
37–48	16 (2.34)	4 (2.30)	7 (9.59)	5 (1.14)	
48–60	3 (0.96)	0 (0)	1 (2.78)	2 (1.06)	
>60	5 (1.21)	1 (0.44)	1 (1.67)	3 (2.44)	
**Sex, boy**	440 (6.71)	77 (3.40)	277 (14.87)	86 (3.53)	0.695^$^
**Year of disease**					<0.001^$^
2009	49 (6.56)	4 (2.76)	42 (15.56)	3 (0.90)	
2010	132 (5.21)	16 (3.59)	66 (11.66)	50 (3.28)	
2011	124 (7.95)	21 (3.01)	99 (16.98)	4 (14.34)	
2012	116 (8.40)	21 (3.97)	81 (13.64)	14 (5.43)	
2013	125 (10.02)	14 (2.77)	95 (23.51)	16 (4.73)	
2014	111 (6.85)	19 (3.54)	55 (12.97)	37 (5.60)	
2015	50 (4.46)	23 (3.97)	19 (8.72)	8 (2.47)	
**HpeV genotype**					*P*_*total*_ < 0.001^$^
					*P*_*singleinfection*_ = 0.187^$^
					*P*_*coinfection*_ < 0.001^$^
HPeV1	396 (3.88)	87 (2.53)	252 (8.24)	57 (1.53)	<0.001^$^
Single infection	120 (1.18)	21 (0.61)	93 (3.04)	6 (0.16)	
Coinfection	276 (2.70)	66 (1.92)	159 (5.2)	51 (1.37)	
HPeV2	1 (0.01)	0 (0)	1 (0.03)	0 (0)	NA
Single infection	0 (0)	0 (0)	0 (0)	0 (0)	
Coinfection	1 (0.01)	0 (0)	1 (0.03)	0 (0)	
HPeV3	46 (0.45)	3 (0.09)	31 (1.01)	12 (0.32)	0.541^#^
Single infection	8 (0.08)	0 (0)	7 (0.23)	1 (0.03)	
Coinfection	38 (0.37)	3 (0.09)	24 (0.78)	11 (0.3)	
HPeV4	86 (0.84)	7 (0.2)	65 (2.12)	14 (0.38)	0.015^#^
Single infection	23 (0.23)	3 (0.09)	20 (0.65)	0 (0)	
Coinfection	63 (0.62)	4 (0.124)	45 (1.47)	14 (0.38)	
HPeV5	18 (0.18)	0 (0)	11 (0.36)	7 (0.19)	1.000 ^#^
Single infection	4 (0.04)	0 (0)	3 (0.1)	1 (0.03)	
Coinfection	14 (0.14)	0 (0)	8 (0.26)	6 (0.16)	
HPeV6	40 (0.39)	8 (0.23)	13 (0.42)	19 (0.51)	0.038^#^
Single infection	3 (0.03)	2 (0.06)	1 (0.03)	0 (0)	
Coinfection	37 (0.36)	6 (0.17)	12 (0.39)	19 (0.51)	
HPeV7	2 (0.02)	0 (0)	1 (0.03)	1 (0.03)	NA
Single infection	0 (0)	0 (0)	0 (0)	0 (0)	
Coinfection	2 (0.02)	0 (0)	1 (0.03)	1 (0.03)	
HPeV8	3 (0.03)	0 (0)	2 (0.07)	1 (0.03)	NA
Single infection	2 (0.02)	0 (0)	2 (0.07)	0 (0)	
Coinfection	1 (0.01)	0 (0)	0 (0)	1 (0.03)	
HPeV10	3 (0.03)	0 (0)	2 (0.07)	1 (0.03)	NA
Single infection	1 (0.01)	0 (0)	1 (0.03)	0(0/0)	
Coinfection	2 (0.02)	0 (0)	1 (0.03)	1 (0.03)	
HPeV14	7 (0.07)	0 (0)	6 (0.20)	1 (0.03)	NA
Single infection	0 (0)	0 (0)	0 (0)	0 (0)	
Coinfection	7 (0.07)	0 (0)	6 (0.20)	1 (0.03)	
Untyped*	115 (1.13)	13 (0.38)	81 (2.65)	21 (0.56)	0.601^#^
Single infection	18 (0.18)	3 (0.09)	13 (0.42)	2 (0.05)	
Coinfection	97 (0.95)	10 (0.29)	68 (2.22)	19 (0.51)	

*Data are n (detection rate%) unless otherwise indicated.*

*^$^Group 1: pediatric patients with respiratory illness; Group 2: pediatric patients with acute diarrhea; Group 3: pediatric patients with Hand foot and mouth disease.*

*The P-value was calculated by comparing Group 1, Group 2, and Group 3 as a general. Proportions may not total 100 because of rounding. There were 10 patients coinfected with two genotypes of HPeVs, including HPeV1-HPeV4 coinfection in 7 patients in Group 2, HPeV1-HPeV4 coinfection in 1 patient in Group 3, HPeV3-HPeV6 coinfection in 1 patient in Group 2, and HPeV1-HPeV5 coinfection in 1 patient in Group 3.*

*^#^Fisher-test, two-sided.*

*^$^Chi-square test, two-sided.*

*Due to genotyping failure.

For Group 1, the 118 HPeV-positive patients were younger than the 3,320 negative patients (*P* = 0.002, Table 2). Compared with those with HPEV single infection, although the coinfection with other respiratory viruses was related to an increased frequency of cough (95.51%), moist rales (92.13%), and dyspnea (17.98%), none of the difference attained significant level. Co-infection of HPeV with other viruses occurred mostly for RSV (31, 34.83%), HRV (29, 32.58%), and PIV (26, 29.21%) (Supplementary Table 3 and Supplementary Figure 1A). Co-infection of HPeV with other two viruses occurred mostly for HpeV-RV-NoV (*n* = 61) (Supplementary Figure 1B). Co-infection of HPeV with other three viruses (RV-NoV-AstV, RV-SaV-NoV, IFV-HBoV-HRV, HAdV-RV-NoV, RSV-IFV-HRV, RSV-PIV-HBoV) were also observed in 10 patients (Supplementary Figure 1C). The interaction analysis between HPeV and other respiratory viruses disclosed significant negative interaction between HPeV and PIV, when HPeV was used as response virus (dependent variable), and PIV was used as the responsive explanatory virus (independent variable) (*P* = 0.046, OR = 0.59, 95% CI = 0.34–0.98) (Supplementary Table 4).

**TABLE 2:** The demographic and clinical characteristics of the HPeV infected patients compared with non-infected.

Variable	HPeV*-*positive			HPeV-negative	*P*-value	
			
	Subtotal	Single-detection	Co-detection		HPeV-positive vs. HPeV-negative	Single-infection vs. co-infection
**Group 1**	**(*n* = 118)**	**(*n* = 29)**	**(*n* = 89)**	**(*n* = 3,320)**		
Age, months (median, IQR)*	6.5(4.0−10.0)	6.0(4.0−10.0)	7.0(4.0−10.0)	9.0(4.0−21.0)	0.002	0.958
Sex, boy (%)^#^	77 (65.25)	17 (58.62)	60 (67.42)	2,186(65.84)	0.859	0.388
Clinical manifestation						
Cough^#^	111 (94.07)	26 (89.66)	85 (95.51)	3,131(94.31)	0.912	0.480
Moist rales^#^	107 (90.68)	25 (86.21)	82 (92.13)	2,985(89.91)	0.86	0.950
Pneumonia^#^	107 (90.68)	25 (86.21)	82 (92.13)	2,985(89.91)	0.785	0.558
Asthma^#^	89 (75.42)	22 (75.86)	67 (75.28)	2,480(74.72)	0.083	1.000
Rhinorrhoea^#^	58 (49.15)	14 (48.28)	44 (49.44)	1,549(46.66)	0.809	0.813
Bronchitis^#^	22 (18.64)	5 (17.24)	17 (19.10)	668 (20.12)	1.000	1.000
Dyspnea^#^	20 (16.95)	4 (13.79)	16 (17.98)	535 (16.11)	0.694	0.823
Rhonchi^#^	8 (6.78)	2 (6.90)	6 (6.74)	399 (12.02)	0.593	0.913
Sore throat^#^	5 (4.24)	1 (3.45)	4 (4.49)	131 (3.95)	0.721	1.000
**Group 2**	**(*n* = 457)**	**(*n* = 137)**	**(*n* = 320)**	**(*n* = 2,602)**		
Age, months (median, IQR)*	8.7(6.3−13.1)	7.8(5.4−11.0)	9.4(6.8−13.2)	10.8(6.7−16.2)	< 0.001	< 0.001
Sex, boy (%)^#^	277 (60.61)	88 (64.23)	189 (59.06)	1,586(60.95)	0.891	0.300
Clinical manifestation						
Diarrhea times ≥ 5^#^	294 (64.33)	80 (58.39)	214 (66.88)	1,636(62.87)	0.551	0.083
Vomiting^#^	169 (36.98)	30 (21.90)	139 (43.44)	1,238(47.58)	< 0.001	< 0.001
Respiratory symptoms^#^	141 (30.85)	40 (29.20)	101 (31.56)	887 (34.09)	0.177	0.616
**Group 3**	**(*n* = 132)**	**(*n* = 10)**	**(*n* = 122)**	**(*n* = 3583)**		
Age, months (median, IQR)*	15.6(12.0−24.8)	11.0(7.7−12.9)	16.1(12.0−25.8)	25.6(17.0−36.0)	< 0.001	0.010
Sex, boy (%)^#^	86 (65.15)	7 (70.00)	79 (64.75)	2,350(65.59)	0.918	1.000
Clinical manifestation						
Neurological complications^#^	59 (44.70)	1 (10.00)	58 (47.54)	1,477(41.22)	0.426	0.049
Respiratory complications^#^	7 (5.30)	0 (0)	7 (5.74)	208 (5.81)	0.808	1.000
Digestive complications^#^	6 (4.55)	1 (10.00)	5 (4.10)	56 (1.56)	0.023	0.383
Circulatory complications^#^	5 (3.79)	0 (0)	5 (4.10)	176 (4.91)	0.556	1.000

*Data are n (%) unless otherwise indicated.*

*Mann-Whitney U-test.

*^#^Chi-square test, two-sided. Single infection refers to the single positive detection of HPeV among all the tested viruses in the group of patients; coinfection refers to the positive detection of HPeV and other tested viruses in the group of patients.*

For Group 2, the 457 HPeV-positive patients were younger compared with 2,602 HPeV-negative patients (*P* < 0.001, Table 1). The 137 patients with HPeV-single infection were younger than the 320 patients with HPeV co-infection (*P* < 0.001). Although less vomiting was observed from HPeV-positive patients than those negative (36.98% vs. 47.58%, *P* < 0.001), HPeV coinfection indeed had higher frequency of vomiting than HPeV single infection (21.90% vs. 43.44%, *P* < 0.001). The co-infection occurred mostly between HPeV and NoV (233, 72.81%), RV (138, 43.13%), and SaV (16, 5.00%) (Supplementary Figure 1 and Supplementary Table 3).

For Group 3, the 132 HPeV-positive patients were younger than the 3,583 HPeV-negative patients (*P* < 0.001, Table 2). The 10 patients with HPeV-single infection were younger than those with co-infection (*P* = 0.010). Compared with the HPeV-negative patients, only digestive system complication was significantly overrepresented in the HPeV-positive patients. More presence of neurological complication was observed from patients with HPeV-coinfection than from those with single infection (47.54% vs. 10.00%, *P* = 0.049). It’s noteworthy in one child with nervous system syndromes, untyped HPeV was detected with all other pathogens tested to be negative. The interaction analysis revealed significant positive interactions between HPeV and EV, when HPeV was used as the responsive virus (dependent variable) and EV was used as the explanatory virus (independent variable) (*P* = 0.015, OR = 2.28, 95% CI = 1.23–4.73) (Supplementary Table 4).

### The Genotype and Genetic Characteristics of HPeV-Positive Patients

**FIGURE 2:**
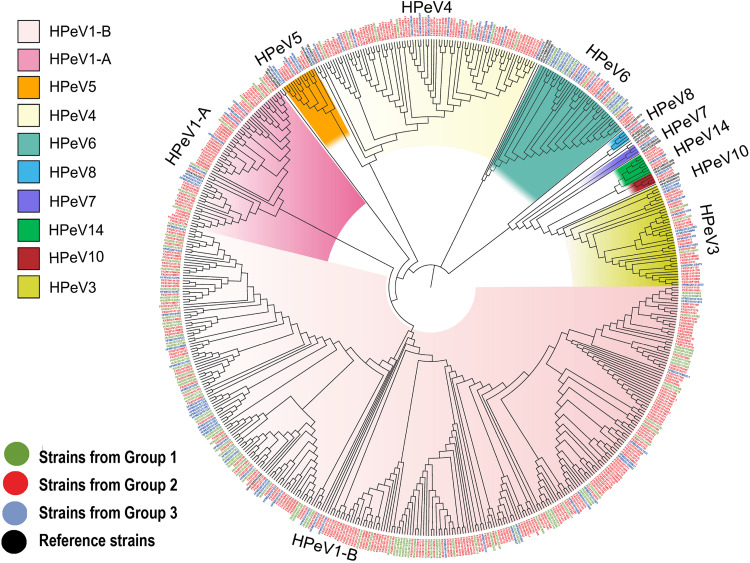
Phylogenetic analysis of all the HPeV strains from our study based on the compete VP1 sequence. The phylogenetic trees were constructed with maximum likelihood method with 1,000 replicates using MEGA 7.0. The strains of black names in the figure were the reference strains (HPeV1A/L02971, HPeV1B/EF051629, HPeV2/AJ005695, HPeV3/AB084913, HPeV4/DQ315670, HPeV5/AM235749, HPeV6/AB252582, HPeV7/EU556224, HPeV8/EU716175, HPeV9/JX219575, HPeV10/GQ402516, HPeV11/JX219574, HPeV12/JX219566, HPeV13/JX219579, HPeV14/MG571809, HPeV15/JX219573, HPeV16/JX219580, HPeV17/KU885089, HPeV18/KY931660, and HPeV19/MH339678). The strains of green names with the beginning of “HUXI” were isolated from patients with Group 1 (patients with acute respiratory tract infection). The strains of red names with the beginning of “FX” were isolated from Group2 (patients with diarrhea). The strains of blue names with the beginning of “HFMD” were isolated from Group3 (patients with hand-foot-mouth disease).

From the 707 HPeV-positive patients, 592 (83.73%) were successfully sequenced for VP1 or VP3/VP1 nucleotide sequences, and 10 genotypes of HPeV were identified. There were 10 patients coinfected with two genotypes of HPeVs, including HPeV1-HPeV4 coinfection in 7 patients in Group 2, HPeV1-HPeV4 coinfection in 1 patient in Group 3, HPeV3-HPeV6 coinfection in 1 patient in Group 2, and HPeV1-HPeV5 coinfection in 1 patient in Group 3. Thus, 602 HPeV genotypes from 592 patients were used for the final analysis. HPeV-1 was the most common genotype (*n* = 396), followed by HPeV4 (*n* = 86), HPeV3 (*n* = 46), and HPeV6 (*n* = 40) (Table 1 and Figure 2). Other uncommon genotypes included HPeV5 (*n* = 18), HPeV14 (*n* = 7), HPeV8 (*n* = 3), HPeV10 (*n* = 3), HPeV7 (*n* = 2), and HPeV2 (*n* = 1). The proportion of genotypes differed among three groups (*P* < 0.001), with HPeV1 and HPeV4 overrepresented among diarrhea patients and HPeV6 overrepresented among HFMD (Figure 1).

**FIGURE 3:**
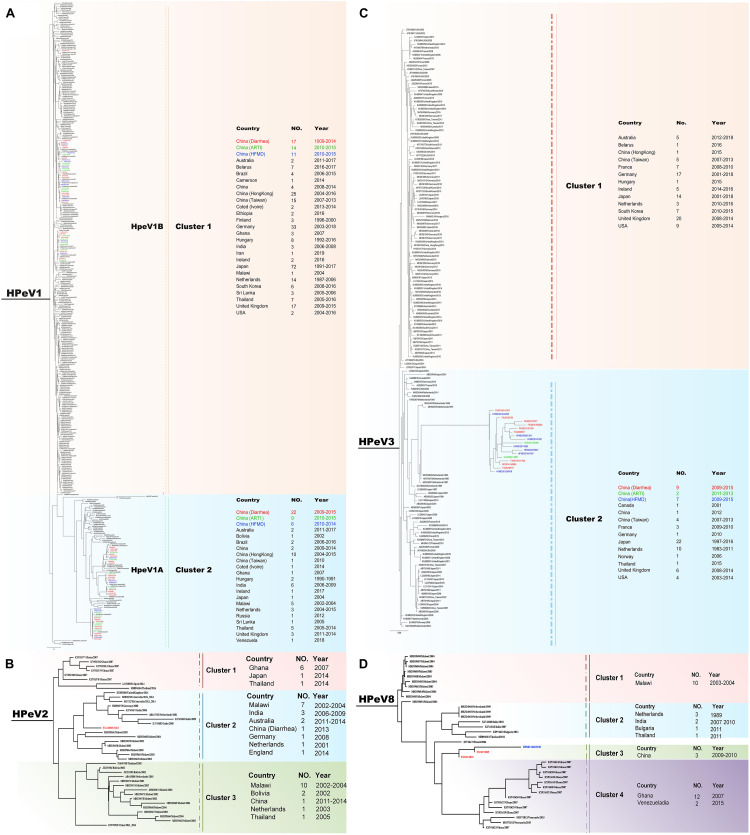
Phylogenetic analysis of all the HPeV1-3 and 8 strains based on the compete VP1 sequence. **(A)** Phylogenetic analysis of representative HPeV1 strains based on the compete VP1 sequence. **(B)** Phylogenetic analysis of all the HPeV2 strains based on the compete VP1 sequence. **(C)** Phylogenetic analysis of representative HPeV3 strains based on the compete VP1 sequence. **(D)** Phylogenetic analysis of all the HPeV8 strains based on the compete VP1 sequence. The phylogenetic trees were constructed with maximum likelihood method with 1,000 replicates using MEGA 7.0. The strains of black names were downloaded from GenBank and others were from our study. The strains of green names with the beginning of “HUXI” were isolated from Group 2 (patients with acute respiratory tract infection). The strains of red names with the beginning of “FX” were isolated from Group 2 (patients with diarrhea). The strains of blue names with the beginning of “HFMD” were isolated from Group 3 (patients with hand-foot-mouth disease).

**FIGURE 4:**
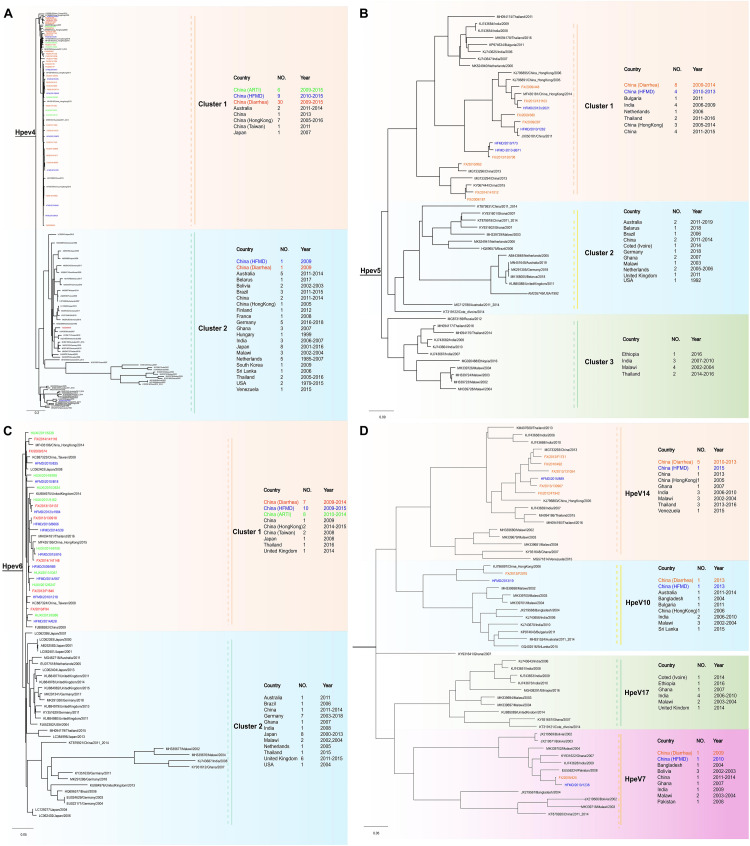
Phylogenetic analysis of all the HPeV4–7, HPeV10, and HPeV14 strains based on the compete VP1 sequence. **(A)** Phylogenetic analysis of representative HPeV4 strains based on the compete VP1 sequence. **(B)** Phylogenetic analysis of representative the HPeV5 strains based on the compete VP1 sequence. **(C)** Phylogenetic analysis of representative HPeV6 strains based on the compete VP1 sequence. **(D)** Phylogenetic analysis of representative HPeV7, HPeV10, and HPeV14 strains based on the compete VP1 sequence. The phylogenetic trees were constructed with maximum likelihood method with 1,000 replicates using MEGA 7.0. The strains of black names were downloaded from GenBank and others were from our study. The strains of green names with the beginning of “HUXI” were isolated from Group 1 (patients with acute respiratory tract infection). The strains of red names with the beginning of “FX” were isolated from Group 2 (patients with diarrhea). The strains of blue names with the beginning of “HFMD” were isolated from Group 3 (patients with hand-foot-mouth disease).

Phylogenetic trees were constructed based on the HPeV VP1 nucleotide sequences obtained from current study and extracted from GenBank. The spatial patterns of the genetic clustering were identified for each genotype. For HPeV1, two branches, HPeV1A and HPeV1B, were formed, and the strains from our three patient groups were located in both HPeV1A and HPeV1B (Figure 3A). For HPeV2, three branches were formed and one strain in 2013 (FX/130905/2013) from Group 2 of our study was located in Cluster 2, together with the strains from Malawi, India, and Australia (Figure 3B). As recently described, 2 HPeV-3 lineages have been identified (Elling et al., 2019) and all the current strains were distributed in Cluster 2.2 together with strains from United States and Japan (Figure 3C). For HPeV4, most current strains were in Cluster 1 (Figure 4A). For HPeV5, our strains were located in Cluster 1 (Figure 4B). For HPeV6, our strains were located in Cluster 1 and Cluster 3 (Figure 4C). The HPeV7 strains from our study were close to Pakistan strain, which collectively formed Cluster 2 (Figure 4D). For both HPeV10 and HPeV14, our strains were close to Hong Kong strain (Figure 4D). The HPeV8 strains from the current study were exclusively located in Cluster 3 (Figure 3D).

The determined clusters were also analyzed for their proportion that differed according to the year of sampling and disease severity. We observed significant difference in the composition of clusters only within HpeV1A along the study years, that altered from 100% of Cluster1.1 in 2009 to 100% of Cluster1.3 in 2015 (*P* < 0.001, Supplementary Table 5 and Supplementary Figure 2). No significant difference was observed for the proportion of clusters regarding the disease severity (Supplementary Tables 5,6 and Supplementary Figures 2, 3).

### The Age Specific Pattern of HPeV Infection

The positive rate of HPeV started to increase after birth to the highest level at the 7–12 months group, then gradually decreased until at a plain level at 25–36 months, with the low level maintained, except with a slight rebound at 37–48 months group for Group 2 (Figure 1B). This was highly similar with the age-specific trend of other detected viral pathogen in Group 1, but not the viral pathogens in Group 2 or 3, where their highest positive rate was observed in the 13–24-month age (Figure 1B). The three most common genotypes, HPeV1, HPeV3, and HPeV4, revealed the same trend that increased after birth to the highest positive level at 7–12 months (Supplementary Table 7).

### The Temporal Pattern of HPeV Infection

During the study period from 2009 to 2015, a biannual cycle of circulation was observed for HPeV infections, i.e., peaks of infections occurred in even-numbered years in Group 1 and Group 3, while in odd-numbered years in Group 2 (Supplementary Figures 4A,B). Similar results were observed for HPeV1 (Supplementary Figure 5A), which showed similar peaks of infection every 2 years. HPeV3 is unusual in exhibiting a biannual cycle of infections, for which the circulation peaked in even-numbered years in Group 2 and Group 3, while the opposite was observed in Group 1 (Supplementary Figures 5C,D). For HPeV4, all three groups were characterized by bi-annual cycles of infections with the circulation peaked in even-numbered years (Supplementary Figure 5E).

For Group 2, an obvious seasonal pattern was observed in that higher positive rates were observed in summer (6.00%, 37.24%) or autumn (5.22%, 23.43%) than those in spring (0.98%, 6.20%) and winter (1.27%, 5.45%) (*P* < 0.001) (Supplementary Table 8 and Supplementary Figure 4C). A major peak was observed during June–October, demonstrated by remarkably higher infection rates, suggesting the possibility of outbreak occurrence during this period. Less obvious seasonal pattern was observed for Group 1 and Group 3, partially owing to the small positive number. The three most common genotypes, HPeV1, HPeV3, and HPeV4 revealed distinct seasonal patterns, with HPeV1 and HPeV4 largely mimicking the seasonal pattern of all HPeV genotypes (Supplementary Figures 5B,F). By contrast, HPeV3 had displayed a biannual cycle of infections, with spring and autumn–winter peaks observed in Group 2, and a single peak observed for Group 1 (in summer) and Group 3 (in winter), respectively (Supplementary Figure 5D).

### Viral Loads and Clinical Phenotypes

Viral loads of three common genotypes, HpeV1, HPeV3, and HPeV4, were measured and related to age, sex, disease severity, and single/co-infection for three types of diseases separately (Supplementary Tables 9–11). It’s disclosed that higher viral loads of HPeV1 were significantly associated with severe diarrhea than the mild diarrhea (*P* = 0.044), and also significantly more associated with HPeV single infection than HPeV-EV coinfection among HFMD patients (*P* = 0.001) (Supplementary Table 9). Higher viral loads of HPeV3 were observed in male than the female patients with acute diarrhea (*P* = 0.035) (Supplementary Table 10). The viral load was also evaluated in corresponding to the days since disease onset, which revealed no significant trend of decreasing as the disease progressed (Supplementary Table 12).

## Discussion

In the current 6-year study, we identified HPeV infection in 6.92% of the pediatric patients with ARTI, acute diarrhea, and HFMD, with comparable levels across the studied years, indicating its persistent circulation in the surveillance region. The current positive rate of 3.55% for HPeV in HFMD patients was comparable with those of previous studies [4.5% in EV negative HFMD patients in China (Li et al., 2019), 2.55% in Japan (Ito et al., 2010)]. While positive detection of HPeV in 14.94% of the pediatric diarrhea was lower than previous studies in Shanghai, China (55%) (Zhong et al., 2011) and in Europe (29.4%) (Zhang et al., 2011), but higher than that studies in Korea (11.1%) (Seo et al., 2015) and Hong Kong (2.3%) (Chiang et al., 2017). However, the comparison among various studies should be explained with caution because the prevalence differed remarkably regarding the patients’ demography, and it also varied depending on the sampling season and residential areas and the detection methods. As has been shown in the current results, the highest infection rate occurred in the age group of 7–12 months and decreased thereafter to a significantly lower level at 24 months, which is consistent with the serological data from Europe and Japan showing that 90% of infants have been infected with at least one HPeV subtype by the age of 2 years (Olijve et al., 2018). However, compared with the commonly seen viruses that were causative of the currently studied diseases, the age with peaking HPeV detection was younger, probably indicating its different manner of acquiring infection at early life.

Studies investigating more than one type of diseases would be helpful in elucidating disease caution of HPeV infections; however, they are scarcely performed. In one study performed on more than one type of patients in Hong Kong (Cabrerizo et al., 2017), 2.3% of the 3911 examined children had HPeV infection. The infected children mostly manifested with upper respiratory tract infection, acute gastroenteritis, and maculopapular rash, however, with no comparison made between patients with different clinical features (Chiang et al., 2017). A Spanish study simultaneously detected HPeV RNA in patients with encephalitis/meningoencephalitis and clinical sepsis, however, with no further genotyping efforts (Cabrerizo et al., 2017). Here by a comparison among case groups of varying clinical spectrum, we found that HPeV1 and HPeV4 were more likely to be identified from acute diarrhea compared with respiratory illness, while HPeV3 was more likely to be identified from acute diarrhea and severe HFMD compared with respiratory illness. This is partially consistent with previous research showing the association between the HPeV1 or HPeV3 infection and acute gastroenteritis (Pham et al., 2011; Zhang et al., 2011).

In the present study, 8 common respiratory viruses in ARTI patients, 5 common viruses in diarrhea patients, and all EVs in HFMD patients were concurrently detected. Only 176 (24.8%) cases had single HPeV infection and the other 75.2% had co-infections. This rate of coinfection was comparable with 60.0% in patients with acute bronchiolitis or pneumonia in Hong Kong (Chiang et al., 2017), 67% in respiratory specimens in the United Kingdom (Harvala et al., 2008), and 71.4% in gastroenteritis patients in North of China (Zhang et al., 2011). The fact that HPeV is often concurrently detected with other viruses has made it difficult to draw conclusions about its pathogenicity merely from PCR-based prevalence studies. Studies comparing HPeV RNA in cases and controls (Zhang et al., 2011) or the detection of the HPeV genome in CSF or other aseptic samples (Boivin et al., 2005; Olijve et al., 2018) can help to settle that issue. Very few clinical studies have evaluated the clinical significance of HPeV viral loads and the findings on this issue so far were inconsistent. In a group of neonates and young infants, higher viral load of HPeV3 in serum was observed in younger age and higher disease severity; however, no such correlation was observed in stool (Aizawa et al., 2016). Other studies reported no correlation between viral load and infection (Zhang et al., 2011) or severity of symptoms (Wildenbeest et al., 2014), both with small sample size and lacking multivariate analysis. Here by performing multivariate analysis to fully consider the effect from demographic factors, we found significant association between higher viral loads of HPeV1 and more severe diarrhea, which indicated a possible pathogenesis role of HPeV1 in the severe diarrhea. To support this finding, we also observed significantly higher positive rate of HPeV in diarrhea than the HFMD patients, when the latter group can act as an internal control without presenting diarrhea, since the same specimen type of stool were tested in both groups. Therefore, according to the “internal symptomatic controls” theory for human bocavirus posed by Allander (2008), a link between HPeV1 and severe diarrhea might be supported by the current findings. For HFMD disease, although HPeV is often co-detected with EV, we indeed determined higher viral loads in HPeV single infection than HPeV-EV coinfection, highlighting the possible role as a cause of HFMD in children. Still studies comparing HPeV in cases and asymptomatic controls may offer further evidence. On the other hand, no decline of viral load was shown as the days from disease onset, indicating a long-term persistence of viral shedding after disease onset. All these findings, taken together, could argue for a causal relationship between HPeV and the clinical disease, which, however, need to be confirmed by further case-control study or serological study, as well as molecular diagnostics of the virus in blood.

Variable results have been reported regarding the seasonality of HPeV infection, revealing a large disparity across studies. In Thailand, the biannual peaks of HPeV infection in the rainy (Jun–Aug) and winter (Nov–Jan) months had been reported (Malasao et al., 2019), while in Spain had been reported in spring and autumn (Cabrerizo et al., 2017). In Italy and Denmark, both EV and HPeV had been suggested to follow a similar seasonal pattern with a single peak during the summer and autumn (Nielsen et al., 2013; Bubba et al., 2017); in Hong Kong, inconsistent seasonal pattern was also observed between HPeV and EV, which occurred in the autumn–winter seasons and in summer and autumn, respectively (Chiang et al., 2017). Meteorological factors are likely to modulate the infection of EV, from direct influence on viral survival to indirect effects on human susceptibility. Despite various reports regarding the seasonality of HPeV infection, few studies have explored the seasonality for HPeV genotypes, and only with the commonly seen genotypes investigated. HPeV1 infections followed a clear seasonal pattern (September–April) in Finland during each individual year from 1995 to 2000 (Tauriainen et al., 2007). With respect to HPeV-3, previous studies had reported the biannual intervals for the infections (Harvala et al., 2011; van der Sanden et al., 2013; Jeziorski et al., 2015). In the current surveillance city, Chongqing, a seasonal peaking of all HPeV was observed in summer and autumn, with the seasonal pattern more obvious in patients with ARTI and acute diarrhea than in those with HFMD. However, three main genotypes (HPeV1, HPeV3, and HPeV4) had shown distinct seasonal peaks, highlighting a bi-annual cycles of all HpeV and two genotypes (HPeV 1 and HPeV 4) with peaks in odd-numbered years, whereas with peaks in even-numbered years HPeV3, as has been reported widely elsewhere (Tapia et al., 2008; Piralla et al., 2012; Schuffenecker et al., 2012; Ghanem-Zoubi et al., 2013; Harvala et al., 2014; Jeziorski et al., 2015; Vergnano et al., 2015). The reasons for this phenomenon underlying the bi-annual cycle of HPeV infections and its much greater frequency in the odd or even years thus remain to be determined. In addition, further investigation is warranted to decipher how the meteorological factors, such as temperature, humidity, and precipitation, might determine the seasonality and periodicity of HPeV infections with wider geographic range.

Importantly, the current study added to the limited sequence analysis on HPeVs, based on a broad collection of HPeVs strains circulating in China. Our study showed that 10 HPeV genotypes and different sublineages belonging to HPeV1, HPeV4, and HPeV6 were co-circulating in China, with two rare genotypes, HPeV7 and HPeV10, reported in China for the first time. Each of the currently determined genotpes, HPeV1, HPeV4, and HPeV6, were grouped into at least two clusters, with no specific temporal or geographic patterns. Strains that belonged to a single genetic lineage of HPeV were also found in both the respiratory and enteric tracts. Considering the wide distribution available from previous publication that reported HPeV1 in Japan and India (Patil et al., 2018; Pham et al., 2019), HPeV4 in Thailand and Netherlands (de Crom et al., 2016a; Malasao et al., 2019), HPeV6 in United Kingdom and Japan (Harvala et al., 2008; Pham et al., 2019), it’s hypothesized that these three HPeVs might be prevalent in children world-wide. Seven other HPeV genotypes (HPeV2, HPeV3, HPeV5, HPeV7, HPeV8, HPeV10, and HPeV14) that were determined in this study were less closely related to the strains found in other regions, possibly indicating spatial differences in the genetic or pathogenic nature of this agent. It is also possible that the observed genetic diversity and the origin of variant strains are limited by the lack of these uncommon HPeV sequences that are available from different regions, which need a larger collection of sequences to be accumulated for analysis. Moreover, the large amounts of untyped HPeVs warrant further sequencing efforts, in order to identify emerging or recombinants HPeV variants with epidemic potential (Pham et al., 2019).

Our study was subject to several limitations. First, we failed to identify a detectable etiology in more than 21% of cases although with multiple tests of viruses. This limitation seemed to be common to surveillance studies with similar study design. Thus, the single infection of HPeV might be a mixed infection with other undetermined pathogens. Second, causal relationships cannot be determined merely from positive detection of HPeVs. This is especially true for HPeV due to its high coinfection rate with other commonly seen pathogens. The clinical spectrum of HPeV and the role that it plays in disease remain to be clarified. Third, although with wide clinical spectrum, the important disease category, sepsis, and meningoencephalitis in neonates and young infants were not included in the study, due to the difficulties in collecting samples from those children undergoing emergency situations.

## Conclusion

In conclusion, the current study based on a comparative analysis of multiple case types and through a long-term surveillance had determined the epidemiological, genetic, and clinical characteristics of HPeV infection in China. The findings have important implications in projecting the expected disease burden and designing interventions aiming to decrease transmission in the vulnerable population. Still, extended surveillance beyond 2015 in wider areas and among various clinical phenotypes might help to acquire an in-depth understanding of its dynamic epidemic features and full spectrum of clinical syndromes. Further investigations are required to determine whether the differentially clustered strains were relevant to their replication, persistence, or pathogenesis.

## Data Availability Statement

The datasets presented in this study can be found in online repositories. The names of the repository/repositories and accession number(s) can be found in the article/Supplementary Material.

## Author Contributions

WL and X-AZ contributed to organizing and supervising the whole study and were also responsible for the funding acquisition. X-AZ, R-QZ, J-JC, YY, XT, and Z-WZ conducted the experiments. Q-BL, Y-NW, H-YZ, P-HZ, and L-QF analyzed the data. X-AZ, R-QZ, and J-JC drafted the manuscript. WL, H-MX, E-ML, and H-SZ mainly contributed to designing the experiments and data analysis. All authors contributed to the article and approved the final manuscript.

## Conflict of Interest

The authors declare that the research was conducted in the absence of any commercial or financial relationships that could be construed as a potential conflict of interest.

## Publisher’s Note

All claims expressed in this article are solely those of the authors and do not necessarily represent those of their affiliated organizations, or those of the publisher, the editors and the reviewers. Any product that may be evaluated in this article, or claim that may be made by its manufacturer, is not guaranteed or endorsed by the publisher.

## Footnotes

1
https://talk.ictvonline.org/

2
http://www.moh.gov.cn/publicfiles/business/htmlfiles/mohyzs/s3586/201004/46884.htm

## Supplementary Material

Supplementary Figure 1 The co-infection between HPeV and other pathogens. **(A)** The co-infection between HPeV and one other pathogen among three groups of pediatric patients. **(B)** The number co-infection between HPeV and two other pathogens. **(C)** The number co-infection between HPeV and three other pathogens.

Supplementary Figure 2 Phylogenetic analysis of all the HPeV1 strains based on the compete VP1 sequence. **(A)** The whole phylogenetic tree of HPeV1. **(B)** The detailed phylogenetic tree for Cluster 2. **(C)** The detailed phylogenetic tree for Cluster 1. The strains of green names with the beginning of “HUXI” were isolated from patients with Group 1 (patients with acute respiratory tract infection). The strains of red names with the beginning of “FX” were isolated from Group 2 (patients with diarrhea). The strains of blue names with the beginning of “HFMD” were isolated from Group 3 (patients with hand-foot-mouth disease).

Supplementary Figure 3 Phylogenetic analysis of all the HPeV3 strains based on the compete VP1 sequence. **(A)** The whole phylogenetic tree of HPeV3. **(B)** The detailed phylogenetic tree for Cluster 1. **(C)** The detailed phylogenetic tree for Cluster 2. The strains of green names with the beginning of “HUXI” were isolated from patients with Group 1 (patients with acute respiratory tract infection). The strains of red names with the beginning of “FX” were isolated from Group 2 (patients with diarrhea). The strains of blue names with the beginning of “HFMD” were isolated from Group 3 (patients with hand-foot-mouth disease).

Supplementary Figure 4 The temporal pattern of HPeV infection. **(A)** The relation between year and HPeV genotypes among all pediatric patients. **(B)** Annual patterns of detected HPeV. **(C)** The relation between season and HPeV detection rate among three groups of pediatric patients. Group 1: patients with acute respiratory tract infection (ARTI), Group 2: patients with acute diarrhea, Group 3: patients with hand, foot and mouth disease (HFMD).

Supplementary Figure 5 The temporal pattern of HPeV1, HPeV3, and HPeV4 infection. **(A)** Annual patterns of HPeV1. **(B)** The relation between year and HPeV1 genotypes among all pediatric patients. **(C)** Annual patterns of detected HPeV3. **(D)** The relation between year and HPeV3 genotypes among all pediatric patients. **(E)** Annual patterns of detected HPeV4. **(F)** The relation between year and HPeV4 genotypes among all pediatric patients. Group 1: patients with acute respiratory tract infection (ARTI), Group 2: patients with acute diarrhea, Group 3: patients with hand, foot and mouth disease (HFMD).
