# Supplementary figures and images for: The Identification and Genetic Characterization of Parechovirus Infection Among Pediatric Patients With Wide Clinical Spectrum in Chongqing, China
Source: Front Microbiol. 2021 Sep 14;12:709849. doi: 10.3389/fmicb.2021.709849 (PMC8477803; doi:10.3389/fmicb.2021.709849)

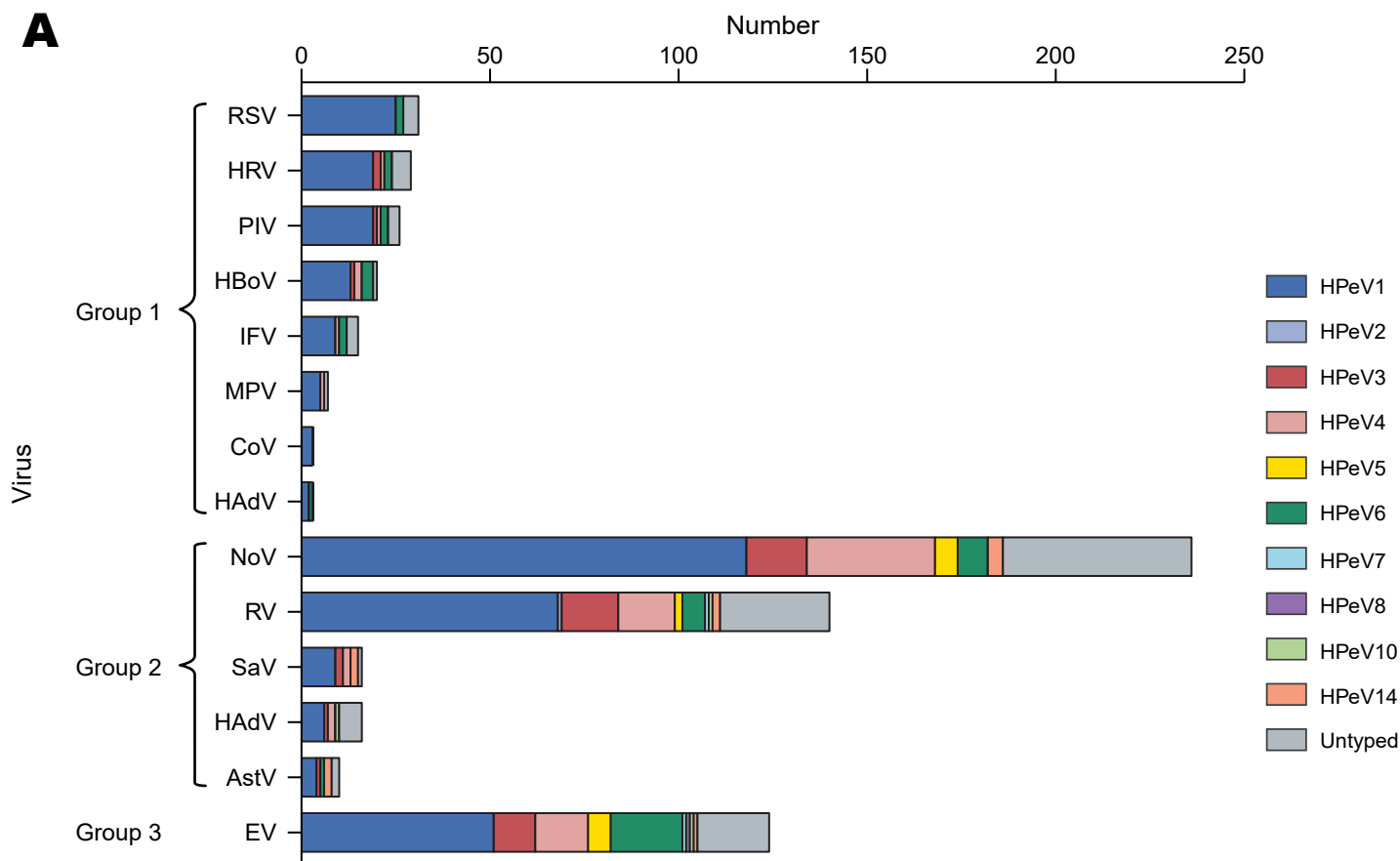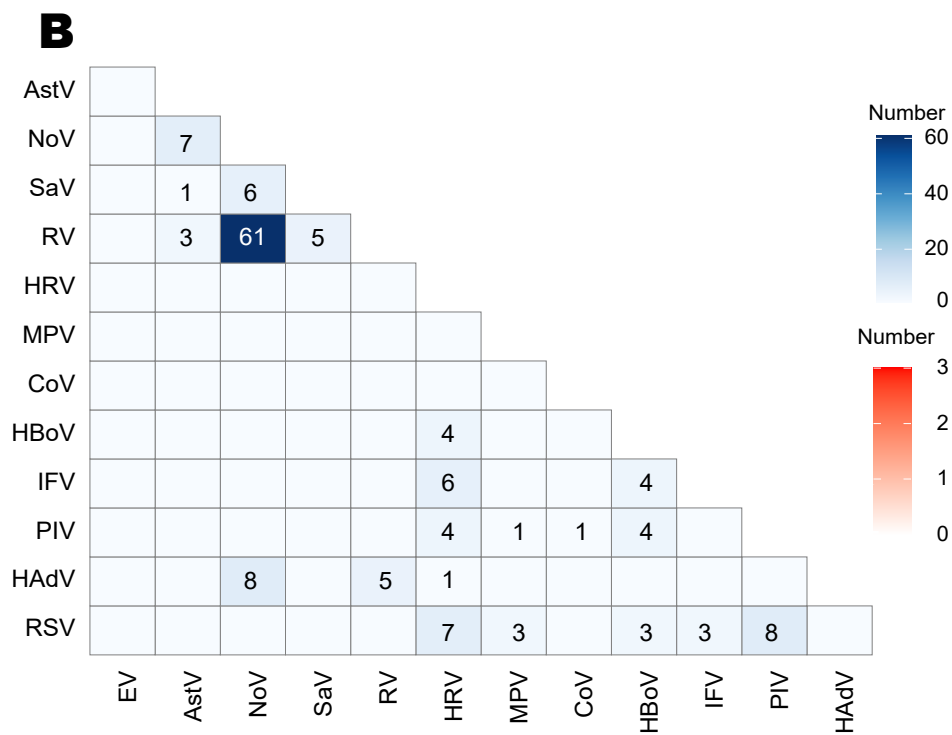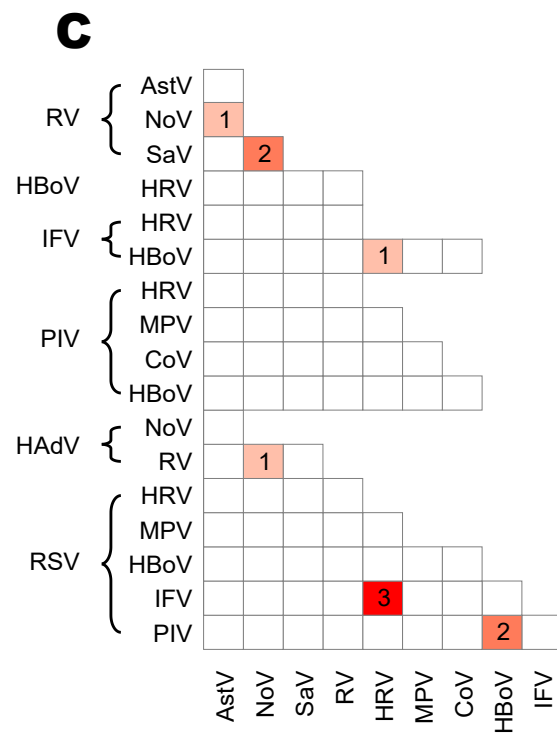

Supplement: Supplementary Figure 1 — The co-infection between HPeV and other pathogens. (A) The co-infection between HPeV and one other pathogen among three groups of pediatric patients. (B) The number co-infection between HPeV and two other pathogens. (C) The number co-infection between HPeV and three other pathogens. [file Image_1.pdf]

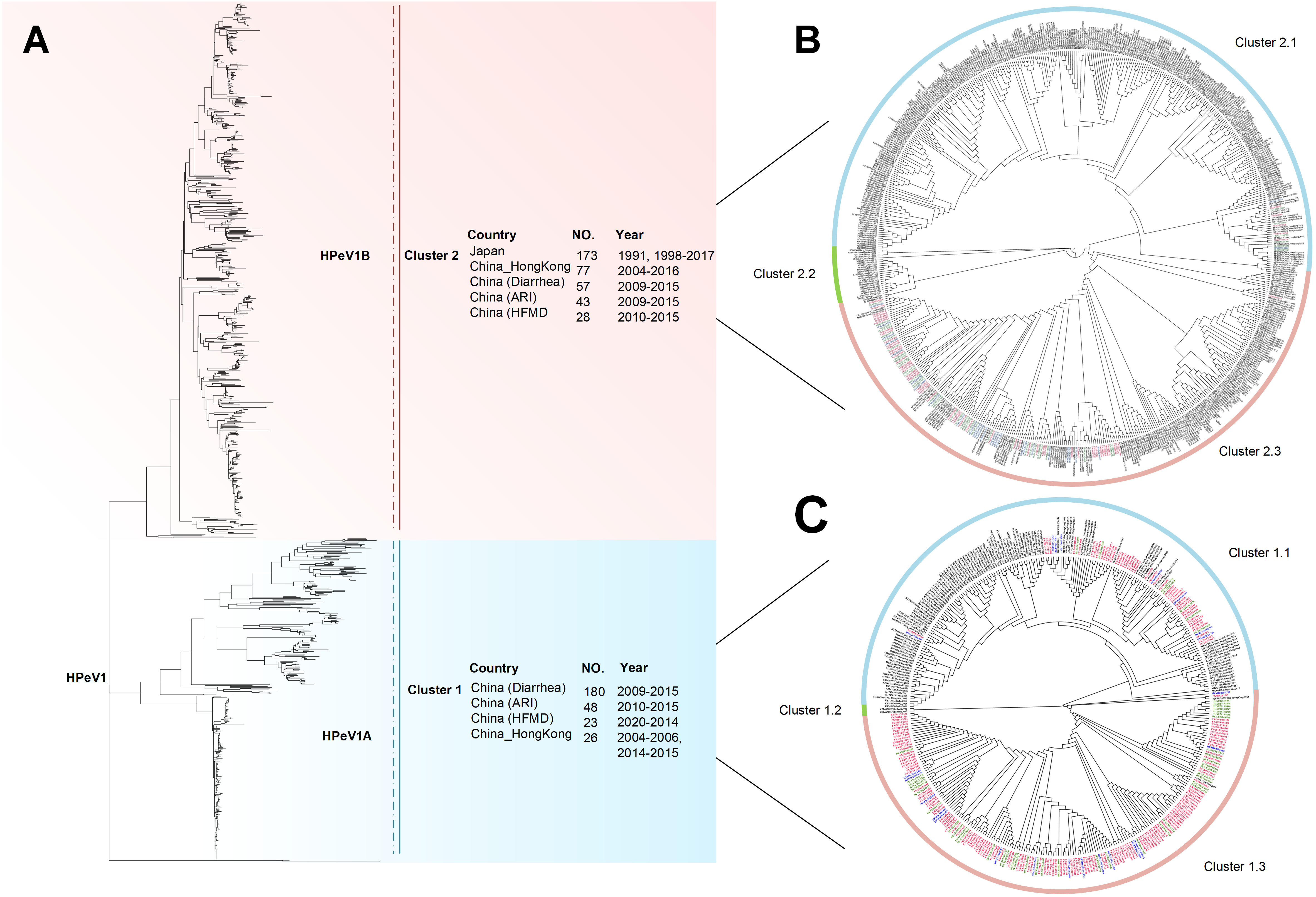

Supplement: Supplementary Figure 2 — Phylogenetic analysis of all the HPeV1 strains based on the compete VP1 sequence. (A) The whole phylogenetic tree of HPeV1. (B) The detailed phylogenetic tree for Cluster 2. (C) The detailed phylogenetic tree for Cluster 1. The strains of green names with the beginning of “HUXI” were isolated from patients with Group 1 (patients with acute respiratory tract infection). The strains of red names with the beginning of “FX” were isolated from Group 2 (patients with diarrhea). The strains of blue names with the beginning of “HFMD” were isolated from Group 3 (patients with hand-foot-mouth disease). [file Image_2.tif]

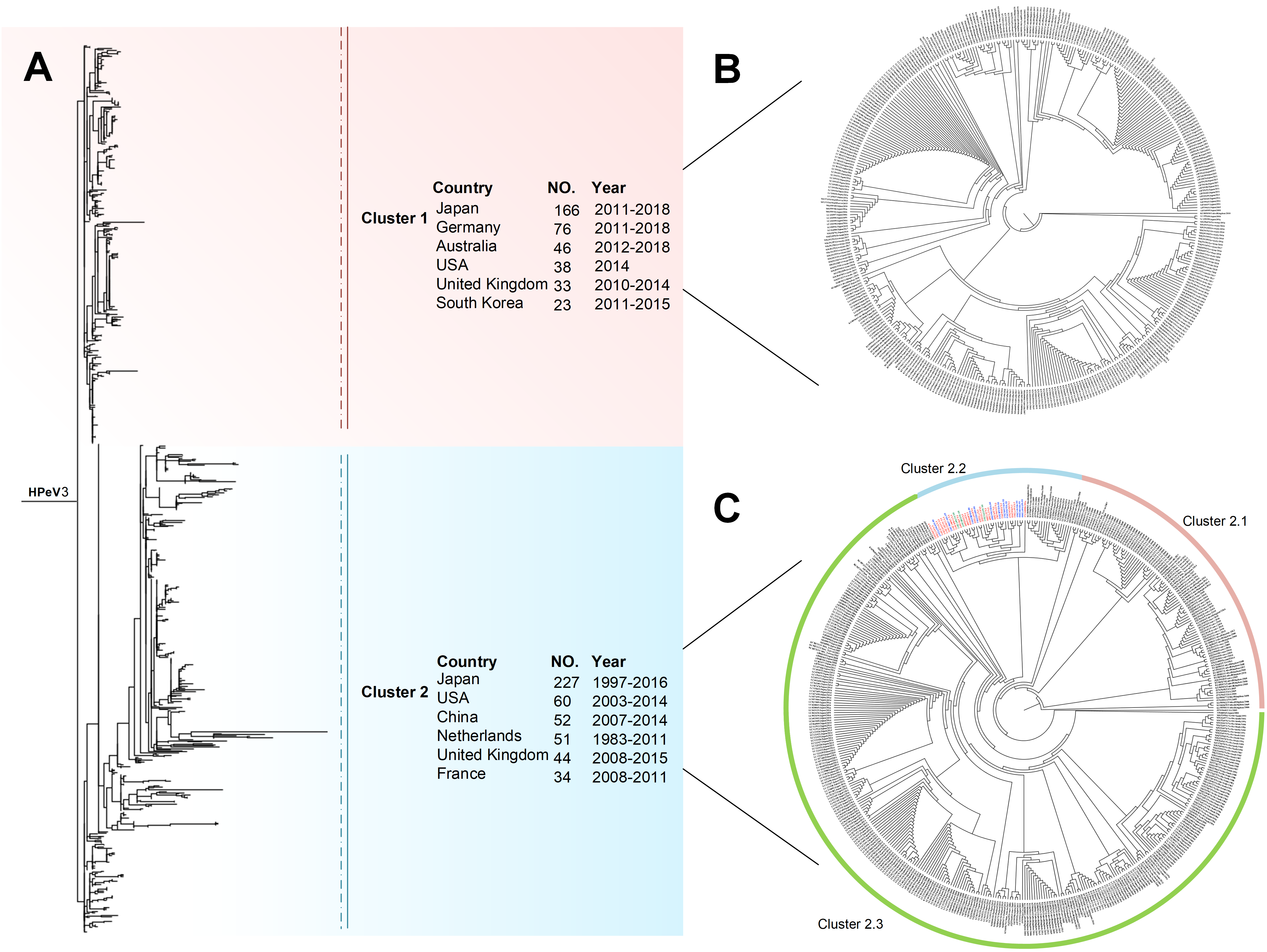

Supplement: Supplementary Figure 3 — Phylogenetic analysis of all the HPeV3 strains based on the compete VP1 sequence. (A) The whole phylogenetic tree of HPeV3. (B) The detailed phylogenetic tree for Cluster 1. (C) The detailed phylogenetic tree for Cluster 2. The strains of green names with the beginning of “HUXI” were isolated from patients with Group 1 (patients with acute respiratory tract infection). The strains of red names with the beginning of “FX” were isolated from Group 2 (patients with diarrhea). The strains of blue names with the beginning of “HFMD” were isolated from Group 3 (patients with hand-foot-mouth disease). [file Image_3.tif]

**A**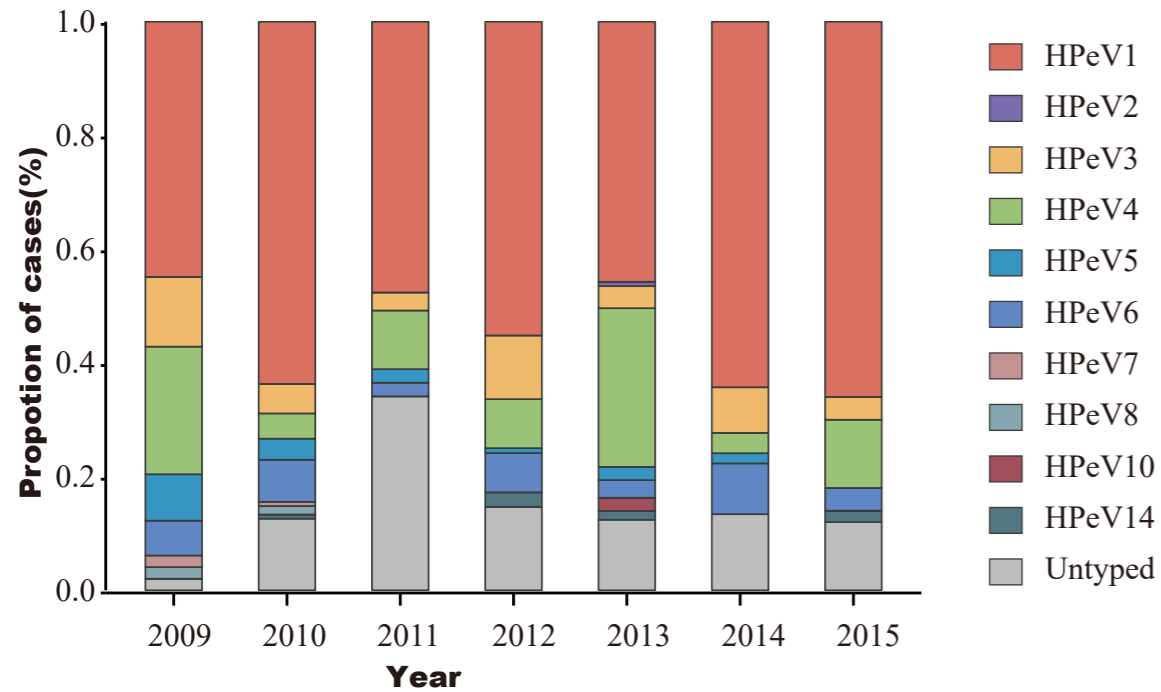**B**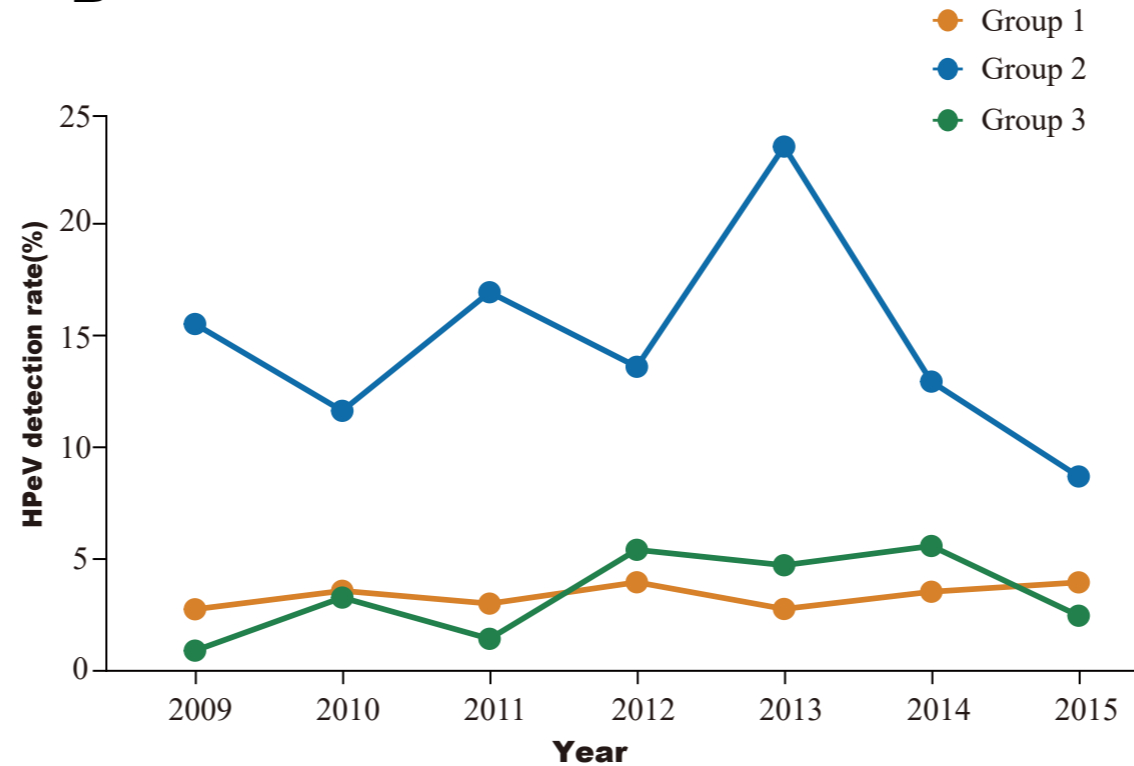**C**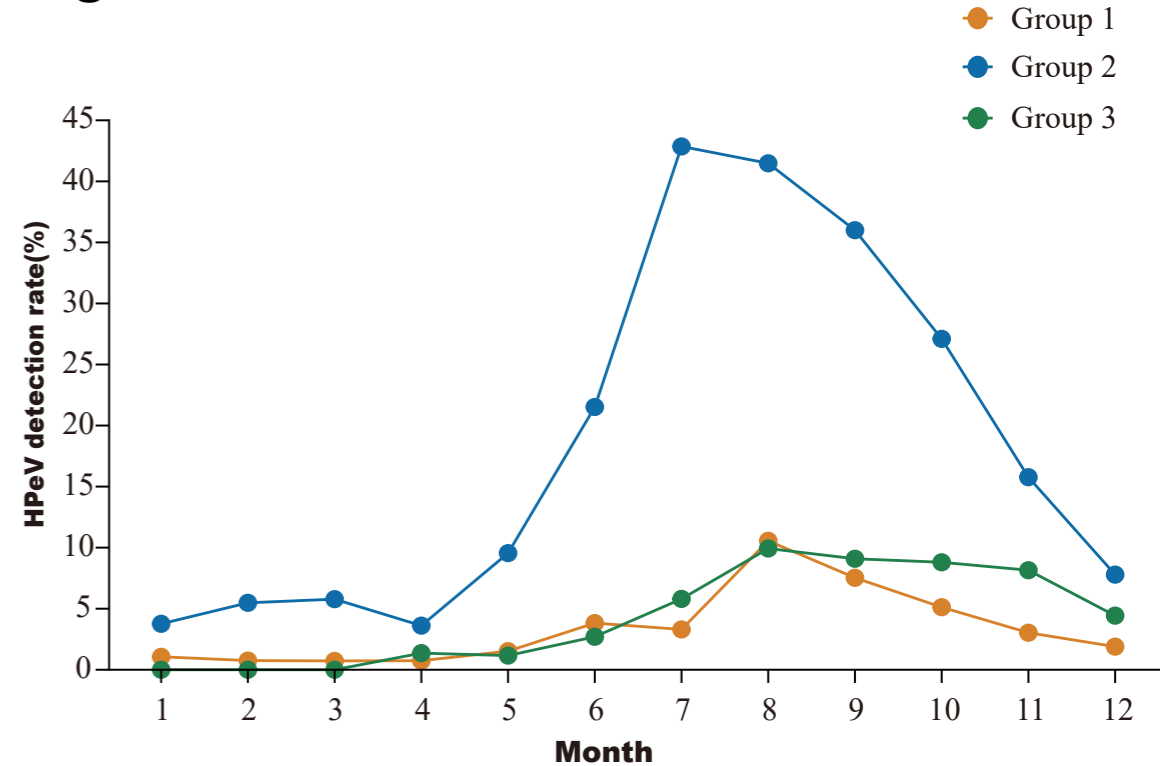

Supplement: Supplementary Figure 4 — The temporal pattern of HPeV infection. (A) The relation between year and HPeV genotypes among all pediatric patients. (B) Annual patterns of detected HPeV. (C) The relation between season and HPeV detection rate among three groups of pediatric patients. Group 1: patients with acute respiratory tract infection (ARTI), Group 2: patients with acute diarrhea, Group 3: patients with hand, foot and mouth disease (HFMD). [file Image_4.pdf]

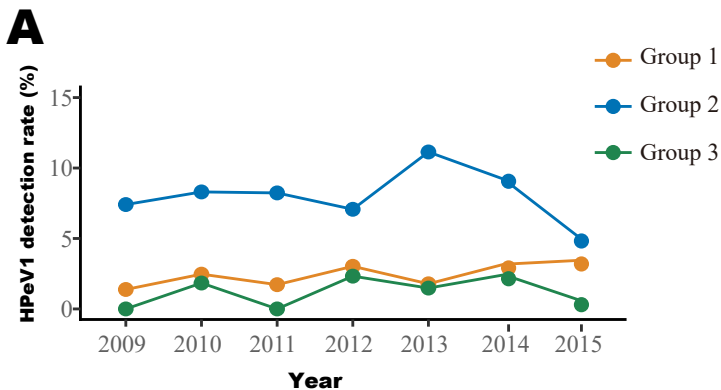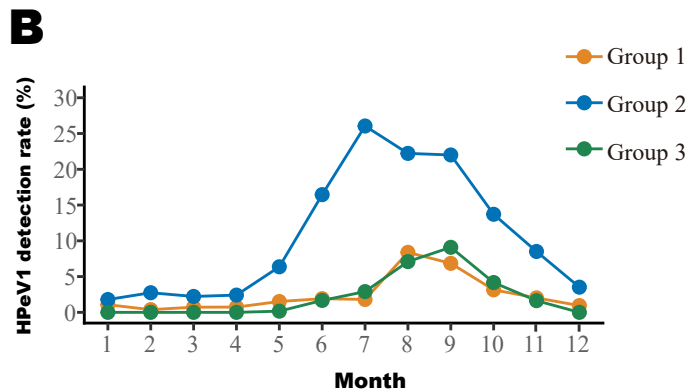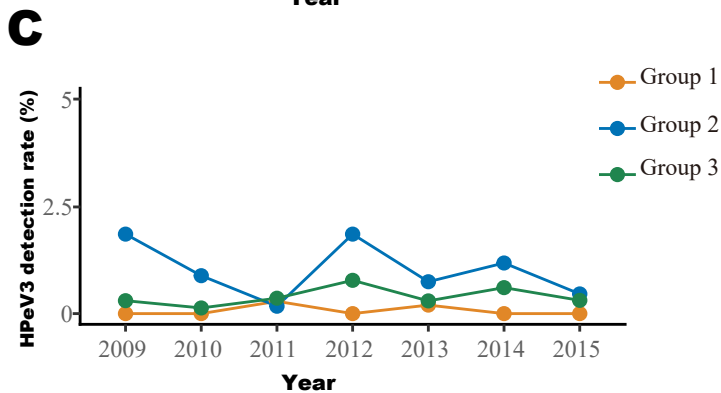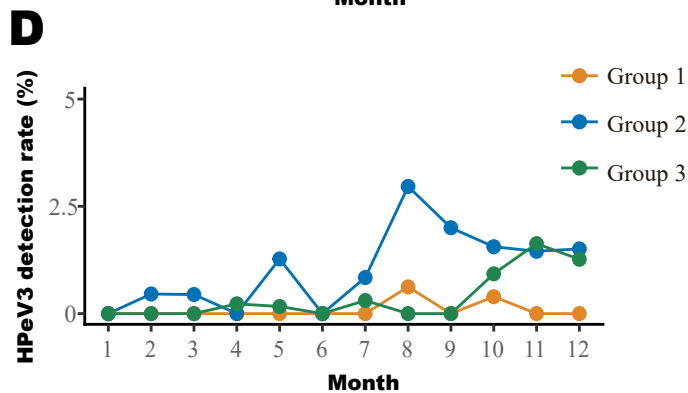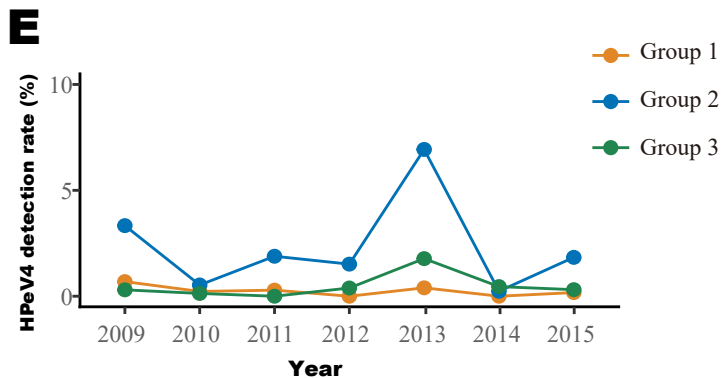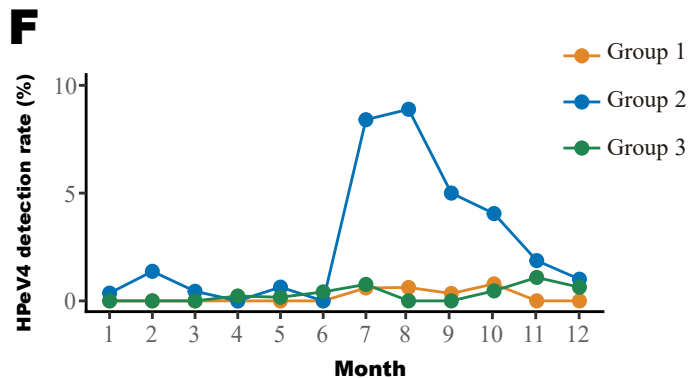

Supplement: Supplementary Figure 5 — The temporal pattern of HPeV1, HPeV3, and HPeV4 infection. (A) Annual patterns of HPeV1. (B) The relation between year and HPeV1 genotypes among all pediatric patients. (C) Annual patterns of detected HPeV3. (D) The relation between year and HPeV3 genotypes among all pediatric patients. (E) Annual patterns of detected HPeV4. (F) The relation between year and HPeV4 genotypes among all pediatric patients. Group 1: patients with acute respiratory tract infection (ARTI), Group 2: patients with acute diarrhea, Group 3: patients with hand, foot and mouth disease (HFMD). [file Image_5.pdf]
