# Supplementary material for: The Identification and Genetic Characterization of Parechovirus Infection Among Pediatric Patients With Wide Clinical Spectrum in Chongqing, China
Source: Front Microbiol. 2021 Sep 14;12:709849. doi: 10.3389/fmicb.2021.709849 (PMC8477803; doi:10.3389/fmicb.2021.709849)
Supplement: Supplementary file 6 [file Data_Sheet_1.docx]

**Supplemental Materials**

**Laboratory detection**

Viral RNA and DNA were extracted using QIAamp^®^MinElute Virus Spin Kits (Qiagen, Hilden, Germany). The cDNA was synthesized by using the SuperScript^®^III First-Strand Synthesis System for reverse transcription polymerase chain reaction (RT-PCR) (Invitrogen, Carlsbad, CA, USA). All samples were screened for HPeV using real-time reverse-transcription polymerase chain reaction (RT-PCR) assay targeting the 5’untranslated region (UTR)(Supplemental Table 2). Quantification of HPeV was performed using qRT-PCR targeting the same gene segments. In brief, plasmids containing known copy number of amplification targets were included in PCR assay to generate a standard curve for quantification of test samples respectively. The copy number of virus was determined by comparison with a serially diluted plasmid standard of known concentration. All samples were quantified in at least duplicate wells. Positive and negative controls were included. Levels ofHPeV RNA concentrations were expressed as copies/mL.All HPeV positive samples were amplified using nested RT-PCR approach for genotyping as previously described (Supplemental Table 1).

**Statistical Analysis**

The interactions between HPeVs and each of the other detected viruses was explored atthe scale of individual hosts. Briefly, the signatures of virus-virus interactions were inferred from the nonrandom patterns of virus mixing among the virus-positive population with a series of binary logistic regression models. ForHPeV, all the remaining pathogens was used as explanatory variables.In addition, we ensured thatpathogens’ associations were not simply explained by independent monthlyfluctuations in the response virus infection risk. To do so, we adjusted thetotal number of infections with the response virus and the totalnumber tested within a 15-d window either side of each (earliest)sample collection date for each individual observation(1)The Holm’s method was used to adjust *P* value for multiple comparisons. R version 3.4.4 was used to conduct these analyses.

**Supplemental Table 1**. The specific primers for genotyping of HPeV.

| ID | Primer name | Forward primer | Reverse primer | Product size(nt) | Gene | **Method** | **References** |
| --- | --- | --- | --- | --- | --- | --- | --- |
| 1 | PeV F1R1 | TNMGNATGGGNTTYTTYCCNAAY | ARTARTCNARYTCR CAYTCYTC | 1203 | VP1 | Nested RT-PCR | (2) |
|  | PeV F2R2 | GAGTTGGACAATGCCATCTAYACNATNTGYG | GTTCCTGTTAGAGCTGTCTTRAANATRTCRTC | 1091 |  |  |  |
| 2 | VP1-parEcho | CCAAAATTCRTGGGGTTC | AAACCYCTRTCTAAATAWGC | 760 | VP1 | Touch-down RT-PCR | (3) |
| 3 | NRZ212/214 | GACAATAGTTTTGAAATNACWATMCC | TAGTGYTTGTARAAACCYCTATCTA | 977 | VP1 | Nested RT-PCR | (4) |
|  | NRZ213/214 | ACTTGGATGAGGAARACWMATGG | TAGTGYTTGTARAAACCYCTATCTA | 938 |  |  |  |
| 4 | HPeV-VP3/VP1 | CATAYTCNTTYTCHACHTGGATGRGGAA | GGNCCATCATCYTGWGCTGANGT | 307 | VP3/VP1 | Touch-down RT-PCR | (5) |
| 5 | F2R2 | TTYTCMACHTGGATGMGGAARAC | DGGYCCATCATCYTGWGCTGA | 434 | VP3/VP1 | Touch-down RT-PCR | (6) |

**Supplemental Table 2. The demographic and clinical characteristics of three groups of pediatric patients.**

| **Characteristics** | **Total** | **Group 1^$^** | **Group 2^$^** | **Group 3^$^** | ***P* value** |
| --- | --- | --- | --- | --- | --- |
|  | **(****n=10212)** | **(n=3438)** | **(n=3059)** | **(n=3715)** |  |
| **Age in months (median, IQR)** | | | | | |
|  | 14 (7.3-29) | 9 (4-20) | 10.3 (6.7-15.4) | 25.2 (16.6-36) | < 0.001***** |
| **Age groups (months)** | | | | |  |
| 0-6 | 2082 (20.39) | 1381 (40.17) | 634 (20.73) | 67 (1.80) | < 0.001^#^ |
| 7-12 | 2366 (23.17) | 797 (23.18) | 1167 (38.15) | 402 (10.82) |  |
| 13-24 | 2732 (26.75) | 512 (14.89) | 917 (29.98) | 1303 (35.07) |  |
| 25-36 | 1622 (15.88) | 255 (7.42) | 172 (5.62) | 1195 (32.17) |  |
| 37-48 | 684 (6.70) | 174 (5.06) | 73 (2.39) | 437 (11.76) |  |
| 48-60 | 314 (3.07) | 90 (2.62) | 36 (1.18) | 188 (5.06) |  |
| >60 | 412 (4.03) | 229 (6.66) | 60 (1.96) | 123 (3.31) |  |
| **Sex, boy (%)** |  |  |  |  |  |
|  | 6562 (64.26) | 2263 (65.82) | 1863 (60.9) | 2436 (65.57) | < 0.001^#^ |
| **Year of disease** |  |  |  |  |  |
| 2009 | 747 (7.31) | 145 (4.22) | 270 (8.83) | 332 (8.94) |  |
| 2010 | 2535 (24.82) | 446 (12.97) | 566 (18.50) | 1523 (41.00) |  |
| 2011 | 1559 (15.27) | 697 (20.27) | 583 (19.06) | 279 (7.51) |  |
| 2012 | 1381 (13.52) | 529 (15.39) | 594 (19.42) | 258 (6.94) | < 0.001^#^ |
| 2013 | 1247 (12.21) | 505 (14.69) | 404 (13.21) | 338 (9.10) |  |
| 2014 | 1621 (15.87) | 536 (15.59) | 424 (13.86) | 661 (17.79) |  |
| 2015 | 1122 (10.99) | 580 (16.87) | 218 (7.13) | 324 (8.72) |  |

Note: **^$^Group**1: pediatric patients with respiratory illness;**Group** 2: pediatric patients with acute diarrhea; **Group**3: pediatric patients with Hand foot and mouth disease. The *P* value was calculated by comparing cohort 1, cohort 2 and cohort 3 as a general. Data are n (%) unless otherwise indicated. Proportions may not total 100 because of rounding.

*Mann-Whitney U test

^♯^Chi-square test, two-sided.

**Supplemental Table 3.**The co-detection number of other viruses with HPeVs.

| **HPeV genotype** | Group**1^$^** | | | | | | | |  | Group**2^$^** | | | | |  | Group**3^$^** |
| --- | --- | --- | --- | --- | --- | --- | --- | --- | --- | --- | --- | --- | --- | --- | --- | --- |
|  | RSV | HRV | PIV | HBoV | IFV | MPV | CoV | HAdV |  | NoV* | RV* | SaV | HAdV* | AstV |  | EV* |
| HPeVs | 31 | 29 | 26 | 20 | 15 | 7 | 3 | 3 |  | 233 | 138 | 16 | 15 | 10 |  | 122 |
| HPeV1 | 25 | 19 | 19 | 13 | 9 | 5 | 3 | 2 |  | 118 | 68 | 9 | 6 | 4 |  | 51 |
| HPeV2 | 0 | 0 | 0 | 0 | 0 | 0 | 0 | 0 |  | 0 | 1 | 0 | 0 | 0 |  | 0 |
| HPeV3 | 0 | 2 | 1 | 1 | 0 | 0 | 0 | 0 |  | 16 | 15 | 2 | 1 | 1 |  | 11 |
| HPeV4 | 0 | 1 | 1 | 2 | 1 | 1 | 0 | 0 |  | 34 | 15 | 2 | 2 | 0 |  | 14 |
| HPeV5 | 0 | 0 | 0 | 0 | 0 | 0 | 0 | 0 |  | 6 | 2 | 0 | 0 | 0 |  | 6 |
| HPeV6 | 2 | 2 | 2 | 3 | 2 | 0 | 0 | 1 |  | 8 | 6 | 0 | 0 | 1 |  | 19 |
| HPeV7 | 0 | 0 | 0 | 0 | 0 | 0 | 0 | 0 |  | 0 | 1 | 0 | 0 | 0 |  | 1 |
| HPeV8 | 0 | 0 | 0 | 0 | 0 | 0 | 0 | 0 |  | 0 | 0 | 0 | 0 | 0 |  | 1 |
| HPeV10 | 0 | 0 | 0 | 0 | 0 | 0 | 0 | 0 |  | 0 | 1 | 0 | 1 | 0 |  | 1 |
| HPeV14 | 0 | 0 | 0 | 0 | 0 | 0 | 0 | 0 |  | 4 | 2 | 2 | 0 | 2 |  | 1 |
| Unknown | 4 | 5 | 3 | 1 | 3 | 1 | 0 | 0 |  | 50 | 29 | 1 | 6 | 2 |  | 19 |

*The sum of HPeV genotypes is different with the number of HPeV because there are 8 cases with 2 HPeV genotypes.

**^$^Group**1: pediatric patients with respiratory illness; **Group** 2: pediatric patients with acute diarrhea; **Group**3: pediatric patients with Hand foot and mouth disease.

Abbreviation: Respiratory syncytial virus, RSV; Human rhinovirus, HRV; Parainfluenza virus, PIV; Human bocavirus, HBoV; Influenza Virus, IFV; Metapneumovirus, MPV; Coronavirus,CoV; Human adenovirus, HAdV; Norovirus, NoV; Rotavirus, RV; Sapovirus, SaV; Astrovirus, AstV; Enterovirus, EV.

**Supplemental Table 4.**Logistic regression modelling of viral interactions between HPeV and others.

| Response virus: HPeV | Odds ratio (95% CI) | | *p*-value* | Holm-adjusted *P* |
| --- | --- | --- | --- | --- |
| Group1 |  | |  |  |
| RSV | 0.96 (0.56, 1.63) | | 0.892 | 1 |
| HAdV | 0.64 (0.15, 1.85) | | 0.469 | 1 |
| PIV | 0.59 (0.34, 0.98)* | | 0.046* | 0.046* |
| IFV | 1.06 (0.56, 1.91) | | 0.85 | 1 |
| HBoV | 1.9 (1.06, 3.28) | | 0.025 | 0.203 |
| CoV | 0.88 (0.21, 2.59) | | 0.84 | 1 |
| MPV | 1.34 (0.52, 2.95) | | 0.504 | 1 |
| HRV | 1.33 (0.78, 2.21) | | 0.282 | 1 |
| Group2 | | | | |
| HAdV | 0.69 (0.37, 1.23) | | 0.232 | 0.926 |
| RV | 0.88 (0.64, 1.2) | | 0.418 | 1 |
| SaV | 1.7 (0.87, 3.18) | | 0.107 | 0.534 |
| NoV | 0.96 (0.67, 1.38) | | 0.828 | 1 |
| AstV | 0.9 (0.41, 1.79) | | 0.781 | 1 |
| Group3 | | | | |
| EV | | 2.28 (1.23, 4.73) | 0.015 | 0.015 |

* HPeV was used as response virus(dependent variable). For each explanatory virus(dependent variable), the logistic regression was additionally adjusted by sex, age, total number of infections with the response virus and the totalnumber tested within a 15-d window either side of each sample collection date for each individual observation (not shown in the table).Abbreviation: Respiratory syncytial virus, RSV; Human rhinovirus, HRV; Human rhinovirus, HRV; Parainfluenza virus, PIV; Human bocavirus, HBoV; Influenza Virus, IFV; Metapneumovirus, MPV; Coronavirus, CoV; Human adenovirus, HAdV; Norovirus, NoV; Rotavirus, RV; Sapovirus, SaV; Astrovirus, AstV; Enterovirus, EV.

**Supplemental Table 5.** The distribution of genotype and Cluster, 2009-2015.

| **Genotype and cluster** | **All** | **Year of disease** | | | | | | | ***P* value**^♯^ |
| --- | --- | --- | --- | --- | --- | --- | --- | --- | --- |
|  |  | **2009** | **2010** | **2011** | **2012** | **2013** | **2014** | **2015** |  |
| **HPeV1A** |  |  |  |  |  |  |  |  | < 0.001 |
| Cluster1.1 | 61 (24.30) | 11 (100) | 20 (37.04) | 10 (18.52) | 9 (18.37) | 3 (7.50) | 8 (30.77) | 0 (0) |  |
| Cluster1.3 | 190 (75.69) | 0 (0) | 34 (62.96) | 44 (81.48) | 40 (81.63) | 37 (92.50) | 18 (69.23） | 17 (100) |  |
| **HPeV1B** |  |  |  |  |  |  |  |  |  |
| Cluster2.1 | 112 (89.60) | 9 (100) | 26 (89.66) | 0 (0) | 8 (88.89) | 17 (94.44) | 35 (87.50) | 17 (85.00) | 0.684 |
| Cluster2.3 | 13 (10.40) | 0 (0) | 3 (10.35) | 0 (0) | 1 (11.11） | 1 (5.56) | 5 (12.50) | 3 (15.00) |  |
| **HPeV2** |  |  |  |  |  |  |  |  | NA |
| Cluster2 | 1 (100) | 0 (0) | 0 (0) | 0 (0) | 0 (0) | 1 (100) | 0 (0) | 0 (0) |  |
| **HPeV3** |  |  |  |  |  |  |  |  | NA |
| Cluster2.2 | 38 (100) | 6 (100) | 7 (100) | 4 (100) | 7 (100) | 5 (100) | 7 (100) | 2 (100) |  |
| **HPeV4** |  |  |  |  |  |  |  |  | 0.109 |
| Cluster1 | 82 (96.47)) | 7 (77.78) | 7 (87.50) | 13 (100) | 10 (100) | 35 (100) | 4 (100) | 6 (100) |  |
| Cluster2 | 3 (3.53) | 2 (22.22) | 1 (12.50) | 0 (0) | 0 (0) | 0 (0) | 0 (0) | 0 (0) |  |
| **HPeV5** |  |  |  |  |  |  |  |  | NA |
| Cluster1 | 13 (100) | 4 (100) | 4 (100) | 0 (0) | 1 (100) | 3 (100) | 1 (100) | 0 (0) |  |
| **HPeV6** |  |  |  |  |  |  |  |  |  |
| Cluster1 | 33 (89.19) | 1 (33.33) | 8 (88.89) | 2 (100) | 8 (100) | 3 (100) | 9 (90.00) | 2 (100) | 0.188 |
| Cluster3 | 4 (10.81) | 2 (66.67) | 1 (11.11) | 0 (0) | 0 (0) | 0 (0) | 1 (10.00) | 0 (0) |  |
| **HPeV7** |  |  |  |  |  |  |  |  | NA |
| Cluster2 | 2 (100) | 1 (100) | 1 (100) | 0 (0) | 0 (0) | 0 (0) | 0 (0) | 0 (0) |  |
| **HPeV8** |  |  |  |  |  |  |  |  | NA |
| Cluster3 | 3 (100) | 1 (100) | 2 (100) | 0 (0) | 0 (0) | 0 (0) | 0 (0) | 0 (0) |  |
| **HPeV10** |  |  |  |  |  |  |  |  | NA |
| Cluster1 | 3 (100) | 0 (0) | 0 (0) | 0 (0) | 0 (0) | 3 (100) | 0 (0) | 0 (0) |  |
| **HPeV14** |  |  |  |  |  |  |  |  | NA |
| Cluster3 | 7 (100) | 0 (100) | 1 (100) | 0 (100) | 3 (100) | 2 (100) | 0 (0) | 1 (100) |  |

Note: Data are n (%) unless otherwise indicated. Proportions may not total 100 because of rounding. Not applicable, NA.

^♯^Fisher-test, two-sided.

**Supplemental Table 6.** The distribution of genotype and Clusteraccording to disease severity.

| **Genotype and cluster** | **All** | | |  | **Group 1^$^** | | |  | **Group 2^$^** | | |  | **Group 3^$^** | | |
| --- | --- | --- | --- | --- | --- | --- | --- | --- | --- | --- | --- | --- | --- | --- | --- |
|  | **Severe** | **Mild** | ***P* value** |  | **Severe** | **Mild** | ***P* value** |  | **Severe** | **Mild** | ***P* value** |  | **Severe** | **Mild** | ***P* value** |
| **HPeV1A** |  |  | 0.096^&^ |  |  |  | 1.000^#^ |  |  |  | 0.994^&^ |  |  |  | 0.744^#^ |
| Cluster1.1 | 26 (31.33) | 35 (20.83) |  |  | 1 (16.67) | 6 (14.63) |  |  | 7 (20.00) | 25 (21.93) |  |  | 16(40.00) | 4(30.77) |  |
| cluster1.3 | 57 (68.67） | 133 (67.51) |  |  | 5 (83.33) | 35 (85.37) |  |  | 28 (80.00) | 89 (78.07) |  |  | 24(60.00) | 9(69.23) |  |
| **HPeV1B** |  |  | 0.237^#^ |  |  |  | 0.564^#^ |  |  |  | 1.000^#^ |  |  |  | 1.000^#^ |
| cluster2.1 | 48 (94.11) | 64 (86.49) |  |  | 5 (100) | 25 (80.65) |  |  | 21 (91.30) | 29 (90.63) |  |  | 22(95.65) | 10(90.91) |  |
| cluster2.3 | 3 (5.89) | 10 (13.51) |  |  | 0 (0) | 6 (19.35) |  |  | 2 (8.70) | 3 (9.38) |  |  | 1(4.35) | 1(9.09) |  |
| **HPeV2** |  |  | NA |  |  |  | NA |  |  |  | NA |  |  |  | NA |
| cluster2 | 0 (0) | 1 (100) |  |  | 0 (0) | 0 (0) |  |  | 0 (0) | 1 (100) |  |  | 0 (0) | 0 (0) |  |
| **HPeV3** |  |  | NA |  |  |  | NA |  |  |  | NA |  |  |  | NA |
| **cluster2.2** | 15 (100) | 23 (100) |  |  | 1 (100) | 2 (100) |  |  | 5 (100) | 15 (100) |  |  | 9 (100) | 6(100) |  |
| **HPeV4** |  |  | 1.000^#^ |  |  |  | 1.000^#^ |  |  |  | 1.000^#^ |  |  |  | 0.275^#^ |
| **cluster1** | 36 (97.30) | 46 (95.83) |  |  | 1 (100) | 6 (100) |  |  | 24 (100) | 39 (97.50) |  |  | 11 (91.67) | 1(50.00) |  |
| **cluster2** | 1 (2.70) | 2 (4.17) |  |  | 0 (0) | 0 (0) |  |  | 0 (0) | 1 (2.50) |  |  | 1 (8.33) | 1(50.00) |  |
| **HPeV5** |  |  | NA |  |  |  | NA |  |  |  | NA |  |  |  | NA |
| **cluster1** | 6 (100) | 7 (100) |  |  | 0 (0) | 0 (0) |  |  | 2 (100) | 4 (100) |  |  | 4 (100) | 3(100) |  |
| **HPeV6** |  |  | 0.602^#^ |  |  |  | 1.000^#^ |  |  |  | 1.000^#^ |  |  |  | 1.000^#^ |
| **cluster1** | 11 (84.62) | 22 (91.67) |  |  | 0 (0) | 8 (100) |  |  | 4 (80.00) | 8 (80.00) |  |  | 7 (87.50) | 6(100) |  |
| **cluster3** | 2 (15.38) | 2 (8.33) |  |  | 0 (0) | 0 (0) |  |  | 1 (20.00) | 2 (20.00) |  |  | 1 (12.50) | 0 (0) |  |
| **HPeV7** |  |  | NA |  |  |  | NA |  |  |  | NA |  |  |  | NA |
| **cluster2** | 2 (100) | 0 (0) |  |  | 0 (0) | 0 (0) |  |  | 1 (100) | 0 (0) |  |  | 1 (100) | 0 (0) |  |
| **HPeV8** |  |  | NA |  |  |  | NA |  |  |  | NA |  |  |  | NA |
| **cluster3** | 3 (100) | 0 (0) |  |  | 0 (0) | 0 (0) |  |  | 2 (100) | 0 (0) |  |  | 1 (100) | 0 (0) |  |
| **HPeV10** |  |  | NA |  |  |  | NA |  |  |  | NA |  |  |  | NA |
| **cluster1** | 1 (100) | 2 (100) |  |  | 0 (0) | 0 (0) |  |  | 0 (0) | 2 (100) |  |  | 1 (100) | 0 (0) |  |
| **HPeV14** |  |  | NA |  |  |  | NA |  |  |  | NA |  |  |  | NA |
| **cluster3** | 4 (100) | 3 (100) |  |  | 0 (0) | 0 (0) |  |  | 2 (100) | 3 (100) |  |  | 1 (100) | 0 (0) |  |

Note: Data are n (%) unless otherwise indicated. Proportions may not total 100 because of rounding. Not applicable, NA.

^&^Chi-square test, two-sided.

^♯^Fisher-test, two-sided.

**^$^Group**1: pediatric patients with respiratory illness; **Group** 2: pediatric patients with acute diarrhea; **Group**3: pediatric patients with Hand foot and mouth disease.

**Supplemental Table 7.**Age specific positive rate of threepredominant HPeV genotypes.

| **Characteristics** | **HPeV1** | | **HPeV3** | | **HPeV4** | | ***P* value** |
| --- | --- | --- | --- | --- | --- | --- | --- |
|  | Positive rate | 95% CI | Positive rate | 95% CI | Positive rate | 95% CI |  |
| **Age group(months) ^#^** | | | | | | | |
| 0-6 | 5.96 (124/2082) | 4.94-6.97 | 0.1 (2/2082) | -0.04-0.23 | 0.77 (16/2082) | 0.39-1.14 | <0.0001 |
| 7-12 | 6.93 (164/2366) | 5.91-7.95 | 0.63 (15/2366) | 0.31-0.95 | 1.65 (39/2366) | 1.14-2.16 | <0.0001 |
| 13-24 | 2.93 (80/2732) | 2.3-3.56 | 0.55 (15/2732) | 0.27-0.83 | 0.73 (20/2732) | 0.41-1.05 | <0.0001 |
| 25-36 | 0.74 (12/1622) | 0.32-1.16 | 0.49 (8/1622) | 0.15-0.83 | 0.12 (2/1622) | -0.05-0.29 | <0.0001 |
| 37-48 | 0.88 (6/684) | 0.18-1.58 | 0.29 (2/684) | -0.11-0.7 | 0.15 (1/684) | -0.14-0.43 | <0.0001 |
| 49-60 | 0 (0/314) | 0-0 | 0.64 (2/314) | -0.24-1.52 | 0 (0/314) | 0-0 | <0.0001 |
| >60 | 0.24 (1/412) | -0.23-0.72 | 0.24 (1/412) | -0.23-0.72 | 0 (0/412) | 0-0 | <0.0001 |

^#^Cochran-Armitage test.

**Supplemental Table 8.**HPeV detection rate in different seasons in three groups of pediatric patients.

|  | **Spring** | **Summer** | **Autumn** | **Winter** | ***P* value** |
| --- | --- | --- | --- | --- | --- |
| All | 1.99 (62/3119) | 9.66 (244/2527) | 13.80 (319/2312) | 3.64 (82/2254) | < 0.001 |
| Group 1**^$^** | 0.98 (8/813) | 6.00 (55/917) | 5.22 (44/843) | 1.27 (11/865) | < 0.001 |
| Group 2**^$^** | 6.20 (34/548) | 37.24 (124/333) | 23.43 (235/1003) | 5.45 (64/1175) | < 0.001 |
| Group 3**^$^** | 1.14 (20/1758) | 5.09 (65/1277) | 8.58 (40/466) | 3.27 (7/214) | < 0.001 |

**^$^Group**1: pediatric patients with respiratory illness; **Group** 2: pediatric patients with acute diarrhea; **Group**3: pediatric patients with Hand foot and mouth disease.

**Supplemental Table 9.** The viral load of HPeV1 in three groups of pediatric patients.

| **Group** | **Viral load* (median, IQR)** | **Adjusted β (95% CI)** | ***P* value** |
| --- | --- | --- | --- |
| **Group1^$^** |  |  |  |
| Age, months |  |  |  |
| ≤6 | 5.01 (4.24-5.90) | 0.19 (-0.20, 0.58) | 0.346 |
| >6 | 4.94 (4.65-5.60) | 1 |  |
| Sex |  |  |  |
| Girl | 4.95 (4.29-6.00) | 1 |  |
| Boy | 4.99 (4.64-5.69) | -0.03 (-0.44, 0.38) | 0.882 |
| Disease severity |  |  |  |
| Mild | 5.04 (4.54-5.80) | 1 |  |
| Severe | 4.69 (4.07-4.99) | -0.56 (-1.24, 0.11) | 0.105 |
| HPeV1 |  |  |  |
| Single infection | 5.14 (4.61-5.67) | -0.03 (-0.50, 0.44) | 0.898 |
| Coinfection | 4.94 (4.45-5.76) | 1 |  |
| **Group2^$^** |  |  |  |
| Age, months |  |  |  |
| ≤12 | 6.02 (4.96-7.11) | 0.14 (-0.21, 0.49) | 0.420 |
| >12 | 5.8 (4.96-6.80) | 1 |  |
| Sex |  |  |  |
| Girl | 5.75 (4.96-6.84) | 1 |  |
| Boy | 6.05 (4.96-7.12) | 0.17 (-0.13, 0.46) | 0.270 |
| Disease severity |  |  |  |
| Mild | 5.76 (4.96-6.74) | 1 |  |
| Severe | 6.2 (4.96-7.19) | 0.31 (0.01, 0.62) | 0.044 |
| HPeV1 |  |  |  |
| Single infection | 6.06 (4.96-6.86) | 0.11 (-0.20, 0.41) | 0.500 |
| Coinfection | 5.86 (4.96-7.03) | 1 |  |
| **Group3^$^** |  |  |  |
| Age, months |  |  |  |
| ≤12 | 5.16 (4.96-5.88) | -0.31 (-0.71, 0.08) | 0.122 |
| >12 | 5.12 (4.96-6.03) | 1 |  |
| Sex |  |  |  |
| Girl | 5.16 (4.96-5.88) | 1 |  |
| Boy | 4.96 (4.96-6.05) | 0.09 (-0.28, 0.45) | 0.651 |
| Disease severity |  |  |  |
| Mild | 4.96 (4.96-6.04) | 1 |  |
| Severe | 5.2 (4.96-5.81) | 0.11 (-0.27, 0.50) | 0.565 |
| HPeV1 |  |  |  |
| Single infection | 6.56 (5.69-7.09) | 1.20 (0.55, 1.85) | 0.001 |
| Coinfection | 5.10 (4.96-5.88) | 1 |  |

**^$^Group**1: pediatric patients with respiratory illness; **Group** 2: pediatric patients with acute diarrhea; **Group**3: pediatric patients with Hand foot and mouth disease.

**Supplemental Table 10**. The viral load of HPeV3 in pediatric patients with acute diarrhea.

| **Group** | **Viral load* (median, IQR)** | **Adjusted β (95% CI)** | ***P* value** |
| --- | --- | --- | --- |
| **Group2^$^** |  |  |  |
| Age, months |  |  |  |
| >12 | 5.52 (4.96-6.35) | 1 |  |
| ≤12 | 6.13 (5.53-6.66) | 0.16 (-0.40, 0.72) | 0.580 |
| Sex |  |  |  |
| Girl | 4.96 (4.96-5.69) | 1 |  |
| Boy | 6.13 (5.52-6.72) | 0.69 (0.08, 1.29) | 0.035 |
| Disease severity |  |  |  |
| Mild | 5.53 (4.96-6.13) | 1 |  |
| Severe | 5.9 (5.17-6.72) | 0.53 (-0.05, 1.12) | 0.088 |
| HPeV3 |  |  |  |
| Coinfection | 5.66 (4.96-6.49) | 1 | 0.430 |
| Single infection | 5.88 (5.54-6.78) | 0.27 (-0.39, 0.94) |  |

*The viral loads were presented as log10 copies/mg specimens.

**^$^Group**1**Group** 2, pediatric patients with acute diarrhea.

**Supplemental Table 11.** The viral load of HPeV4 in pediatric patients with acute diarrhea.

| **Group** | **Viral load* (median, IQR)** | **Adjusted β (95% CI)** | ***P* value** |
| --- | --- | --- | --- |
| **Group2^$^** |  |  |  |
| Age, months |  |  |  |
| >12 | 4.96 (4.96-6.54) | 1 |  |
| ≤12 | 6.23 (5.51-6.82) | 0.48 (-0.2, 1.16) | 0.170 |
| Sex |  |  |  |
| Girl | 4.96 (4.96-6.62) | 1 |  |
| Boy | 6.45 (5.58-6.99) | 0.55 (-0.02, 1.13) | 0.066 |
| Disease severity |  |  |  |
| Mild | 6.34 (5.02-6.82) | 1 |  |
| Severe | 6.01 (4.96-6.77) | -0.19 (-0.76, 0.39) | 0.522 |
| HPeV4 |  |  |  |
| Coinfection | 6.17 (4.96-6.78) | 1 |  |
| Single infection | 6.29 (5.61-7.02) | 0.17 (-0.42, 0.76) | 0.568 |
|  |  |  |  |

*The viral loads were presented as log10 copies/mg specimens.

**^$^Group**1**Group** 2, pediatric patients with acute diarrhea.

**Supplemental Table 12.** The viral load of HPeV1, HPeV3, HPeV4 according to delay between onset and hospital admission for three groups of pediatric patients.

| **Group** | **Viral load* (median, IQR)** | ***P* value^#^** |
| --- | --- | --- |
| **Group 1^$^** |  |  |
| HPeV1 Delay, day |  |  |
| ≤6 | 5.05 (4.45-5.64) | 0.804 |
| >6 | 4.96 (4.4-5.52) |  |
| HPeV3 Delay, day |  |  |
| ≤8 | 4.45 (4.45-4.45) | 1.000 |
| >8 | 3.96 (3.96-3.96) |  |
| HPeV4 Delay, day |  |  |
| ≤7 | 5.44 (5.24-5.63) | 1.000 |
| >7 | 5.27 (4.61-5.92) |  |
| **Group 2^$^** |  |  |
| HPeV1 Delay, day |  |  |
| ≤5 | 6.05 (5.03-7.07) | 0.727 |
| >5 | 5.78 (4.81-6.75) |  |
| HPeV3 Delay, day |  |  |
| ≤3 | 5.64 (4.89-6.38) | 0.631 |
| >3 | 6.12 (5.65-6.59) |  |
| HPeV4 Delay, day |  |  |
| ≤5 | 6.17 (5.15-7.19) | 0.559 |
| >5 | 6.06 (5.49-6.63) |  |
| **Group 3^$^** |  |  |
| HPeV1 Delay, day |  |  |
| ≤3 | 5.03 (4.47-5.58) | 0.940 |
| >3 | 5.11 (4.73-5.49) |  |
| HPeV3 Delay, day |  |  |
| ≤3 | 5.48 (5.08-5.89) | 0.724 |
| >3 | 5.47 (5.14-5.8) |  |
| HPeV4 Delay, day |  |  |
| ≤4 | 5.16 (4.76-5.55) | 0.637 |
| >4 | 5.45(4.98-5.91) |  |

**^$^Group1: pediatric patients with respiratory illness; Group 2: pediatric patients with acute diarrhea; Group 3: pediatric patients with Hand foot and** mouth disease.

**^#^**Mann-Whitney U test, two sides.

*The viral loads were presented as log10 copies/mg specimens.

**References**

1. S. Nickbakhsh, C. Mair, L. Matthews, R. Reeve, P. C. D. Johnson, F. Thorburn, B. von Wissmann, A. Reynolds, J. McMenamin, R. N. Gunson and P. R. Murcia: Virus-virus interactions impact the population dynamics of influenza and the common cold. *Proc Natl Acad Sci U S A* (2019) doi:10.1073/pnas.1911083116

2. M. M. Alam, A. Khurshid, S. Shaukat, M. S. Rana, S. Sharif, M. Angez, N. Nisar, M. Naeem and S. S. Zahoor Zaidi: Human parechovirus genotypes -10, -13 and -15 in Pakistani children with acute dehydrating gastroenteritis. *PLoS One*, 8(11), e78377 (2013) doi:10.1371/journal.pone.0078377

3. K. Sano, H. Hamada, S. Hirose, K. Sugiura, S. Harada, M. Koizumi, M. Hara, H. Nishijima, M. Taira, A. Ogura, T. Ogawa and J. I. Takanashi: Prevalence and characteristics of human parechovirus and enterovirus infection in febrile infants. *Pediatr Int*, 60(2), 142-147 (2018) doi:10.1111/ped.13467

4. H. Harvala, C. P. Sharp, E. M. Ngole, E. Delaporte, M. Peeters and P. Simmonds: Detection and genetic characterization of enteroviruses circulating among wild populations of chimpanzees in Cameroon: relationship with human and simian enteroviruses. *J Virol*, 85(9), 4480-6 (2011) doi:10.1128/jvi.02285-10

5. L. Bubba, M. Martinelli, L. Pellegrinelli, V. Primache, E. Tanzi, E. Pariani and S. Binda: A 4-year Study on Epidemiologic and Molecular Characteristics of Human Parechoviruses and Enteroviruses Circulating in Children Younger Than 5 Years in Northern Italy. *Pediatr Infect Dis J*, 36(1), 13-19 (2017) doi:10.1097/inf.0000000000001344

6. H. Harvala, I. Robertson, E. C. McWilliam Leitch, K. Benschop, K. C. Wolthers, K. Templeton and P. Simmonds: Epidemiology and clinical associations of human parechovirus respiratory infections. *J Clin Microbiol*, 46(10), 3446-53 (2008) doi:10.1128/jcm.01207-08
